# *Chenopodium quinoa* Willd. and *Amaranthus hybridus* L.: Ancestral Andean Food Security and Modern Anticancer and Antimicrobial Activity

**DOI:** 10.3390/ph16121728

**Published:** 2023-12-15

**Authors:** Juan Carlos Romero-Benavides, Evelyn Guaraca-Pino, Rodrigo Duarte-Casar, Marlene Rojas-Le-Fort, Natalia Bailon-Moscoso

**Affiliations:** 1 Departamento de Química, Facultad de Ciencias Exactas y Naturales, Universidad Técnica Particular de Loja, Loja 110108, Ecuador; ecguaraca@utpl.edu.ec; 2 Maestría en Alimentos, Facultad de Ciencias Exactas y Naturales, Universidad Técnica Particular de Loja, Loja 110108, Ecuador; 3 Tecnología Superior en Gestión Culinaria, Pontificia Universidad Católica del Ecuador—Sede Manabí, Portoviejo 130103, Ecuador; rduarte@pucesm.edu.ec (R.D.-C.); erojas@pucesm.edu.ec (M.R.-L.-F.); 4 Facultad de Ciencias de la Salud, Universidad Técnica Particular de Loja, Loja 110108, Ecuador; ncbailon@utpl.edu.ec

**Keywords:** ancestral foods, phytochemicals, anticancer activity, antimicrobials, neglected crops, nutritional composition

## Abstract

The species *Chenopodium quinoa* Willd. and *Amaranthus hybridus* L. are Andean staples, part of the traditional diet and gastronomy of the people of the highlands of Colombia, Ecuador, Peru, Bolivia, northern Argentina and Chile, with several ethnopharmacological uses, among them anticancer applications. This review aims to present updated information on the nutritional composition, phytochemistry, and antimicrobial and anticancer activity of Quinoa and Amaranth. Both species contribute to food security due to their essential amino acid contents, which are higher than those of most staples. It is highlighted that the biological activity, especially the antimicrobial activity in *C. quinoa*, and the anticancer activity in both species is related to the presence of phytochemicals present mostly in leaves and seeds. The biological activity of both species is consistent with their phytochemical composition, with phenolic acids, flavonoids, carotenoids, alkaloids, terpenoids, saponins and peptides being the main compound families of interest. Extracts of different plant organs of both species and peptide fractions have shown in vitro and, to a lesser degree, in vivo activity against a variety of bacteria and cancer cell lines. These findings confirm the antimicrobial and anticancer activity of both species, *C. quinoa* having more reported activity than *A. hybridus* through different compounds and mechanisms.

## 1. Introduction

Our perception of staples is centered around Middle Eastern cereals, often leaving aside other staples domesticated and grown elsewhere, often by cultures and civilizations later overrun by invaders, such as ancestral grains, many of which became neglected or underutilized crops.

The *Amaranthaceae* family provides several crops of interest: the economically relevant spinach (*Spinacia oleracea* L.) and sugar beet (*Beta vulgaris* L.) and the ancestral South American pseudocereals quinoa (*Chenopodium quinoa* Willd.) and amaranth (*Amaranthus hybridus* L.). Both species are experiencing renewed interest from alimentary and biological activity perspectives [1]. Interest in Quinoa as a hardy crop began in the first half of the twentieth century, and its popularity exploded in the 1980s [2]. Quinoa became an upper-class staple in the USA, the United Kingdom and other countries in the 2010s, which drove prices up [3]; amaranth expanded as a leaf vegetable and saw a similar price increase [4,5]. Both quinoa and amaranth are considered ‘superfoods’—“foods beyond the diet, but before the drugs” [6]. This status has increased commercial and scientific interest in both species as foods and nutraceuticals.

Antibiotic-resistant bacteria (ARB) are bacteria that are not controlled or killed by antibiotics. Of concern are multidrug-resistant (MDR) bacteria, which are a growing public health problem that needs to be solved [7]. Antimicrobial-resistant infections are predicted to cause around 10 million deaths per year and a total GDP loss of the order of USD 100 trillion by 2050 if effective actions are not implemented, particularly in developing countries where the risk is higher [8,9]. “New agents displaying innovative chemistry and modes of action are desperately needed worldwide to tackle the public health menace posed by antimicrobial resistance” [10]. There is a constant search for new drugs that relies on traditionally used plants as a starting point [11].

Cancer is a group of diseases characterized by unchecked cell growth [12]. It is the second leading cause of death in high-income countries, behind cardiovascular disease [13]. In 2020, there were 19.3 million new cancer cases and 10 million deaths due to this disease [14]. In spite of these numbers, during the last decade, an encouraging general decrease in death rates due to cancer was seen, except for liver cancer in both sexes and lung cancer in women, for which death rates have increased [15]. This general reduction can be attributed, at least in part, to the development and approval of new treatments against the disease [16]. An estimated 25% of the treatments developed in the last decade are derived from natural products, mostly of plant origin, with increasing numbers of products isolated from fungi, bacteria and other kingdoms [17]. The search for active secondary metabolites—and other products, such as proteins and peptides—is fundamental in making progress against the disease.

Infectious diseases are a risk factor for cancer: Hepatitis B and C, Epstein–Barr and human papilloma viruses, *Helicobacter pylori*, and *Streptococcus bovii* are examples. Also, molecular mechanisms of cancer and infection spread are alike and are countered as foreign invaders by the immune system. Thus, antimicrobial and anticancer activities of species, extracts and compounds may be linked, and this is why both activities are reviewed together [18,19].

Quinoa and amaranth are related food crops with interesting nutritional profiles and similar modes of consumption; both are neglected crops due to the arrival of the Spaniards in the late fifteenth century and hold great potential from alimentary and medicinal points of view, appearing together in more than 450 publications according to a Dimensions search [20]. Given this, they are reviewed together.

**Figure 1:**
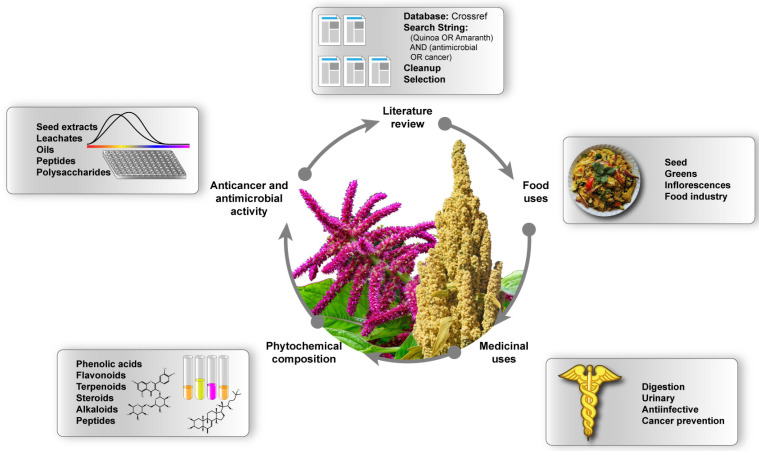
Review flow chart.

There is renewed interest in validating the ethnopharmacological uses of ancestral plant species, and Quinoa and Amaranth have shown promise in several medical uses—cancer prevention and treatment as well as antimicrobial activity featuring prominently. Although there are several excellent reviews on these species, we found a lack of specific anticancer and antimicrobial activity reviews. The aim of this work is to compile and summarize the research about the anticancer and antimicrobial activity of Quinoa and Amaranth to identify the most active and promising lines of work and envision future research directions (Figure 1).

## 2. Method

A systematic literature review was performed following the SPAR-4-SLR (Scientific Procedures and Rationales for Systematic Literature Reviews) protocol [21]. The procedure is outlined in Table 1.

Data were obtained from the Dimensions web application that queries the Crossref database, which is more comprehensive than Scopus and Web of Science [22]. The search string was (Quinoa OR Amaranth) AND (antimicrobial OR cancer) in title and abstract, without time limitations and including only articles and book chapters. The first dataset consisted of 221 documents from 166 sources and a total of 959 authors. The dataset was organized by Dimensions rank, with fields of research (ANZSRC 2020) as the secondary key [23]. The document exclusion criteria are listed in Table 2. All articles not excluded were included.

## 3. Background

The genus *Chenopodium* has worldwide distribution and 130 accepted species [24]. *Chenopodium quinoa* was domesticated about 7000 years ago in the area surrounding Lake Titicaca, an area currently shared by Peru and Bolivia in South America, whence it spread to diverse landscapes which correspond to modern-day Ecuador, Perú, Bolivia, Argentina and Chile [2,25]. At least two other independent domestication events are considered: Chile, 3000 BC, and Bolivia, 750 BC [26].

**Figure 2:**
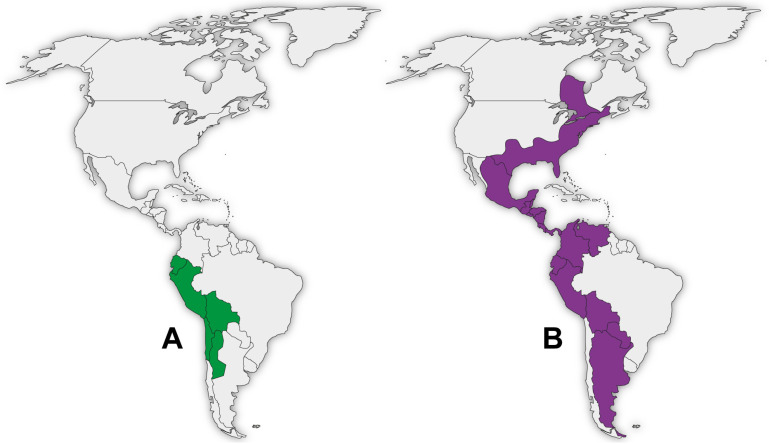
Distribution by country of *C. quinoa* (**A**) in green and *A. hybridus* (**B**) in purple.

The cosmopolitan genus *Amaranthus*, with worldwide distribution, has 95 accepted species. The earliest domestication findings of *Amaranthus hybridus* date from 7000–8000 years ago in what is now Argentina in South America [24]. The species is native to Argentina, Belize, Bolivia, Brazil, Colombia, Costa Rica, Ecuador, El Salvador, Guatemala, Honduras, Mexico, Nicaragua, Panamá, Paraguay, Peru, Uruguay and Venezuela [27,28]. The distribution of *C. quinoa* and *A. hybridus* is shown in Figure 2.

*C. quinoa* is an annual, dicotyledonous plant belonging to the *Amaranthaceae* family. Its height varies from 30 cm to 2.20 m, and it has a taproot with a size range from 0.8 to 1.5 m. The stem that connects to the root is cylindrical, while in the area where it joins the leaves and branches, it becomes angular. Its color varies with maturity. During the flowering period, it appears green, whereas in its mature state, it has a cream or pinkish color. The leaves have two structures: the petiole and the blade. The petiole originates directly from the stem. The blade is larger in the foliage than in the inflorescence and is triangular or lanceolate in shape. The fruit is a spheroidal, lenticular or conical achene, measuring 1.5 to 3 mm. It consists of a pericarp and seed. The pericarp is attached to the seed and contains saponins, which impart a bitter taste unless rinsed, although it has been reported that monomeric saponins present an umami flavor profile and that the bitterness could correspond to phenolic compounds [29]. The seeds are composed of the perisperm, embryo and endosperm. The embryo contains two cotyledons and the radicle, both contributing up to 30% of the seed’s weight. The perisperm is the storage tissue and is primarily made up of starch granules [30]. *C. quinoa* is of particular interest due to its high stress resistance, which may provide food security in the face of advancing desertification [31].

*A. hybridus* is an annual, dicotyledonous plant also in the *Amaranthaceae* family. Its height can reach up to 65 cm. The root is a taproot with secondary and tertiary roots that can grow up to 40 cm in length, which helps it tolerate water scarcity. The stem is cylindrical, longitudinally grooved and glabrous and can be green, purple or reddish in color. The leaves are oval-shaped, simple, alternate or opposite, with wavy edges, glabrous, and green during the growth period, turning violet or purple with prominent veins in maturity. The inflorescences are terminal or axillary, forming a panicle, and have an intense purple color. The flowers are unisexual and pistillate with five unequal tepals. The fruit corresponds to a pyxidium, which releases the seeds when mature [32].

Both species have been used since pre-agricultural times, and have long been cultivated following ancestral, sustainable methods that enable production in conditions of high altitude, drought, salinity and frost and which hold valuable lessons for the current climate emergency [33,34,35].

## 4. Nutritional Properties and Food Uses

Quinoa and amaranth are staples, neglected for centuries after the Spanish conquest, that are currently experiencing a revival [4,36,37]. The protein content of both species is variable but higher than that of cereals (12–19%) and lower than that of legumes and provides all nine essential amino acids in high-quality protein [38,39]. The fat content of both species is higher than that of cereals and abundant in essential unsaturated fatty acids. A comparison of example proximal compositions of quinoa and amaranth seeds is shown in Table 3.

Quinoa protein is high in essential amino acids, with notably high levels of lysine, methionine, histidine, isoleucine and cysteine, which are important in human health [40]. Amaranth seed protein is of high quality, with all nine essential amino acids, and is particularly high in lysine and tryptophan [41]. Amaranth leaf protein is also high-quality, with 36% of its weight in essential amino acids [42].

The seed oils of both species are rich in linoleic and linolenic (18:2 and 8:3) acids, with a ω-6-to-ω-3 proportion close to the recommendation for a healthy diet [38,43]. In addition, both species contain terpenoids of interest: squalene, isoprenoids and phytosterols [44].

The seeds and leaves of both species are vitamin and mineral sources: *C. quinoa* provides vitamins A, B_1_, B_2_, B_3_, B_5_, B_6_ B_9_, B_12_, C and E [45], while *A. hybridus* is a source of vitamins A, B_1_, B_2_, B_3_, B_6_, C and E [46]. *C. quinoa* is a source of calcium, phosphorus, magnesium, potassium, sodium, copper, zinc and iron, while *A. hybridus* provides calcium, phosphorus, magnesium, potassium, sodium, copper, iron, manganese and zinc [47]. In general, amaranth has a higher mineral content than quinoa [4].

In 1996, the FAO declared quinoa a promising crop to address human nutrition problems due to its nutritional and beneficial properties, and 2013 was declared ‘The International Year of Quinoa’ [48]. In the Andean highlands, quinoa is used to prepare soups, refreshing beverages and salads, which are consumed as part of breakfast, lunch and dinner. It is also often combined with legumes to improve the diet quality of preschool and schoolchildren populations [49].

Seeds are the main plant organ used as food for both quinoa and amaranth. Also, tender stems and leaves are used as greens in salads, or are blanched, steamed, boiled or fried. Inflorescences are also edible: the inflorescences of quinoa are used as seasoning and those of amaranth as medicinal and coloring agents in the popular traditional Ecuadorian drinks *horchata* and *colada morada* [50,51,52].

Both quinoa and amaranth seeds are highly nutritious, are naturally gluten-free and have a low glycemic index (GI). All these properties make them desirable in modern dietary trends [53,54], and recipe books combining traditional and modern recipes have been published [55].

Modern uses of both species are varied. The main domestic uses for quinoa are stews, desserts and drinks, and for Amaranth are salads and stews and, in African countries, sauces [56,57,58]. There are currently several processed food products made from, or supplemented with, flour and protein concentrates of both species: pasta, bread, breakfast cereals, sausages, snacks, biscuits, weaning foods, plant-based milks and yogurt, often bearing functional and ethical claims [59,60,61,62]. Quinoa and, to a lesser extent, Amaranth have been included in the haute cuisine offerings of Andean countries and Mexico since the second decade of the 21st century and are offered in Michelin-starred restaurants [63,64,65]. The species are also being used both as substrates and active extracts in edible films [66,67]. Also, the consumption of sprouts is encouraged for the enhanced nutrient bioavailability and antinutrient reduction [68]. Both species are also used in animal feed supplementation in cattle and to improve egg quality in laying hens [69,70].

Among the functional and nutraceutical uses of both species, probiotic-rich drinks from red quinoa have shown potential for restoring the gut microbiota, while the modification of quinoa proteins improves their functional properties and digestibility for the development of improved plant-based foods, and their antioxidant and other activities have promoted the development of several health food products [41,71,72,73].

## 5. Medicinal Uses

Besides their food uses, quinoa and amaranth have been traditionally appreciated as medicinal species by the Andean plateau people [74]. Quinoa seed, stems, fruits and leaves are traditionally used for diverse medicinal purposes, as shown in Table 4.

Amaranth is a popular medicinal plant frequently found in Andean markets [76]. The whole plant is used as medicine, with specific uses for leaves and inflorescences [77]. There is evidence that *A. hybridus* has a beneficial effect on the gut microbiota that underlies its ethnomedical uses concerning digestive system conditions [78]. It is also worth mentioning that *A. hybridus* and other *Amaranthus* species can be poisonous to cattle, exhibiting nephrotoxicity [79]. Traditional medicinal uses for *A. hybridus* are shown in Table 5.

Among the several uses of both species, anti-infective, antiabscess and other uses related to antimicrobial activity, as well as anticancer and chemopreventive uses, are of interest to this work.

## 6. Phytochemical Composition

The composition of *C. quinoa* and *A. hybridus* is diverse. Table 6 shows the main compound families found in both species [78,83].

Twenty-nine phenolic acid derivatives have been identified in Quinoa: sixteen benzoic acid derivatives and thirteen cinnamic acid derivatives [84]. Kongdang et al. reported gallic, ellagic, chlorogenic, and caffeic acids and derivatives in *A. hybridus* [78].

Thirty-five flavonoid compounds have been identified in Quinoa: four flavones, twenty-one flavonols, three flavanones, three flavanols and five estrogenic isoflavonoids [85]. Six flavonols have been isolated from Amaranth, mostly from methanolic leaf extract [86,87].

Fifteen monoterpenoids have been identified in *C. quinoa* essential oils, as well as the sesquiterpene caryophyllene. Bitter triterpenoid saponins and their aglycones are the reason why quinoa seeds must be rinsed before preparation. Ten hederagenins; four spergulagenic acids; five serjanic acids; ten phytolaccagenic acids; two gypsogenins; two 3β, 23, 30 trihydroxy oleano 12-en-28-oic acid analogues; and eleven oleanoic acids, glycated and aglycones, have been isolated, which exhibit antifungal, anti-inflammatory and cytotoxic properties [88,89,90]. Tetra- and pentacyclic terpenoids, and also meroterpenoids, have been identified in *C. quinoa* [91]. The carotenoid compounds lutein and β-carotene were identified in *A. hybridus* leaves [92]. Ten C-27, fourteen C-28 and seven C-29 steroids with antiobesity, antiangiogenic, collagenase inhibition, antioxidant, antidiabetic and anti-inflammatory activity have been identified in *C. quinoa* [91].

The biologically active, nitrogen-containing betalain pigments amaranthine and isoamaranthine, amongst others, have been identified in *A. hybridus* and other *Amaranthus* species [93]. *C. quinoa* varieties exhibit distinctive coloring, caused in part by betaxanthins and betacyanins, such as amaranthin, betanin, dopaxanthin, miraxanthin V (4) and indicaxanthin [94].

There is much interest concerning bioactive proteins and peptides from both species. Lunasin is a 43-amino acid-residue peptide present in quinoa with various health-promoting properties, including antioxidant, anti-inflammatory, hypocholesterolemic and anticancer activities [95]. Lunasin-like peptides are present in amaranth [96]. Detailed lists of phytochemicals from *C. quinoa* and *A. hybridus* are listed in Tables S1 and S2.

## 7. Biological Activity

### 7.1. Overview

**Figure 3:**
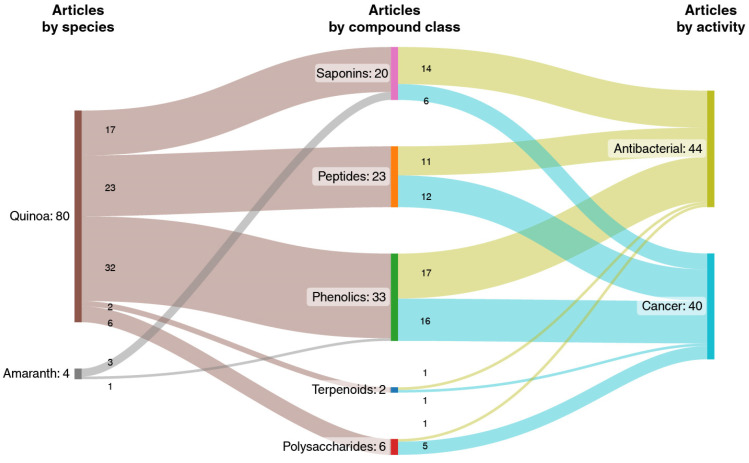
Published articles on antibacterial and anticancer activity of compound classes from *C. quinoa* and *A. hybridus*.

According to the literature review, *C. quinoa* and *A. hybridus* present both antibacterial and anticancer activity, with more reported results for *C. quinoa* than for *A. hybridus*. The research on the main compound families concerning their antibacterial and anticancer activity in both species is shown in Figure 3.

These results show that research on quinoa phytochemicals is more abundant than that on *A. hybridus* phytochemicals. The bioactivity of *A. hybridus* extracts has been shown, but there has been little research progression from extracts to phytochemicals. The most-studied compound classes are phenolics (39%), peptides (27%), saponins (24%), polysaccharides (7%) and terpenoids (2%). Phenolic compounds, saponins and peptides are the most-studied antimicrobials, while phenolics, peptides and saponins are the most-studied compounds with respect to their anticancer activity.

### 7.2. Antimicrobial Activity

**Figure 4:**
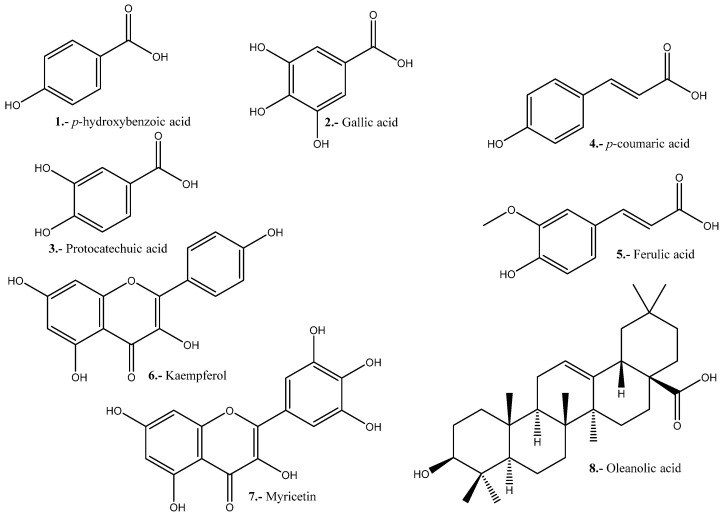
Quinoa compounds with antimicrobial activity.

The antimicrobial activity of *C. quinoa* is due to several compound families, mainly phenolics. Compounds present in extracts of quinoa seeds, leaves, roots and inflorescences exhibit antimicrobial activity [97,98]. Examples of such activity are shown in Table 7 and Figure 4.

Even though Amaranth pigments and other phytochemicals possess antimicrobial activity, various solvent extracts of Amaranth leaves and stems are reported to exhibit weak or no antibacterial activity on the strains tested [107,108,109,110,111]. A mouthwash containing ethyl acetate extract of *A. hybridus* leaves has been reported to be effective against *Streptococcus mutans* [112]. Other species from the genus do exhibit antibacterial and antifungal activity [113,114].

### 7.3. Anticancer Activity

Phenolic compounds, unsaponifiable lipids, terpenoids, peptides and polysaccharides are reported as possessing anticancer activity in both Quinoa and Amaranth [95,115,116]. Antioxidant compounds and activity are associated with cancer prevention but do not imply chemopreventive or anticancer activity and can even interfere with chemotherapeutics [117].

#### 7.3.1. Anticancer Activity of *C. quinoa*

Extracts, powders and oils from quinoa seed, leaves and bran have been reported as possessing anticancer activity. Quinoa leaves have been reported as chemopreventive supplements due to their antioxidant potential and high bioavailability of active compounds [118]. Quinoa bran has been reported as a source of bioactive compounds with antioxidant, antidiabetic, anti-inflammation, antibacterial and anticancer properties [116,119].

Shen et al. evaluated the anticancer activity of the oil from the seeds of the white (WSO), red (RSO) and black (BSO) varieties in HCT116 cells (human colon carcinoma) [120]. The application of BSO was more efficient than WSO and RSO, their IC_50_ values being 281.9, 381.3 and 647.4 µg/mL, respectively. Based on these results, the inhibitory effects of BSO at concentrations of 0, 62, 5, 125 and 250 µg/mL were tested for 36 h, and apoptotic states at concentrations of 62.5–250 µg/mL which indicated significant inhibition were observed. In addition, a change in morphology was observed, since the cells in culture appeared fusiform and retractile and the untreated cells presented a homogeneous, polygonal shape, confirming that BSO can induce significant apoptosis in HTC 116 cells. In quinoa inflorescence extracts, several compounds with anticancer activity have been found, among them 4-hydroxy-benzaldehyde, 1*H*-Indole-3-carboxaldehyde, methyl hexadecanoate and ((6*E*,10*E*,14*E*,18*E*)-2,6,10,15,19,23-hexamethyltetracosa-1,6,10,14,18,22-hexaen-3-ol) [97].

Phenolics in quinoa partially account for both antimicrobial and cytotoxic activities. Stikić et al. found that the Puno cultivar has a higher phenolic content and cytotoxic activity than the Titicaca cultivar against HCT 116 human colorectal cancer cells [121]. Of note is the difference in ferulic acid content between the cultivars. Quinoa seed powder was found to possess an IC_50_ of 14.6 μg/mL against HEPG2 liver carcinoma cells, attributable to their phytic acid, polyunsaturated fatty acid and phenolic contents [122].

Quinoa proteins are reported as having anticancer activity. The cytotoxicity of quinoin—a Type 1 Ribosome-Inactivating Protein (RIP)—extracted from quinoa seeds was investigated in two primary glioblastoma (GBM) cell lines: NULU and ZAR, and in continuous human glioblastoma cells (U87Mg) [123]. The IC_50_ values of quinoin were determined at 24, 48 and 72 h at concentrations 0.001, 0.1, 1.0, 2.5 and 5.0 µM using the tetrazolium dye (MTT) assay. Additionally, the GBM, NULU and ZAR cell lines were treated with 1 µM Temozolomide plus 2.5 nM quinoin for 24, 48 and 72 h. The study showed that quinoin strongly reduced the growth of glioblastoma cells and that the IC_50_ of primary and continuous GBM cells does not depend on the time in contact with quinoin. Furthermore, primary cells treated with quinoin in combination with temozolomide (TMZ—a chemotherapeutic used in the treatment of glioblastoma) were more sensitive to the treatment; therefore, the authors highlight that quinoin could represent a novel tool for the therapy of glioblastoma and a possible adjuvant for the treatment of the disease in combination with TMZ. Lunasin is a protein present in quinoa that also has reported anticancer activity [95].

The anticancer activity of quinoa polysaccharide (CQP) composed of glucose and galacturonic acid units was evaluated by Hu et al. on two types of cancer (SMMC 7721 liver cancer and MCF 10 A breast cancer) using the in vitro MTT assay [124]. For the study, they used normal human liver cells (L02) and normal breast epithelial cells (MCF10A) as controls. Human liver cancer cells (SMMC 7721) and human breast cancer cells (MCF-7) were seeded on plates treated with CQP concentrations of 12.5 and 200 µg/mL and cultured at 37 °C for 24 and 48 h. MTT was added in an amount of 5 mg/mL to each well. After the incubation time, the microplates were measured in a reader at an absorbance of 570 nm. The cell viability rate was estimated in relation to the absorbance given by each well of the samples treated with CQP and the absorbance of the untreated wells. The IC_50_ values of CQP in SMMC 7721 cells after 24 h and 48 h were 121.4 µg/mL and 53.4 µg/mL. The IC_50_ values of CQP in MCF-7 cells after 24 h and 48 h were 83.48 µg/mL and 64.67 µg/mL, with no effect on normal cells. Therefore, the results are indicative that the polysaccharides of *Ch. quinoa* present anticancer activity. On the other hand, quinoa saponin-rich extracts and their hydrolysates were found to have no activity against human colorectal cancer cells [125].

Aqueous leachates from germinating quinoa seeds contain 20-Hydroxyecdysone, a hormonal steroid that has exhibited in vitro anticancer activity against human non-small cell lung cancer cells [126,127].

#### 7.3.2. Anticancer Activity of *A. hybridus*

*Amaranthus hybridus*, along with other species of the genus, shows chemoprotective activity, which is supported by its phytochemical composition. Phenolics, peptides, carotenoids and other compounds have shown activity in this regard [128,129].

Adewale and Olorunju showed a hepatoprotective effect against arsenite-induced cancer in rats [130]. The effect is also evident against aflatoxin and fumonisin damage in rat hepatic cells and is attributed to antigenotoxic phytochemicals and minerals: “phenolics, carotenoids, folic-, linolenic-, linoleic and palmitic acids, as well as calcium, magnesium, iron, zinc, and selenium” [131]. This is in line with other chemoprotective effects attributable to antioxidant properties, for example, against imidacloprid [132]. Due to the selenium content of *A. hybridus* leaves, an aqueous extract was tested for cytotoxicity against the MDA-MB-231 cancer cell line, but no activity was detected [133].

**Figure 5:**
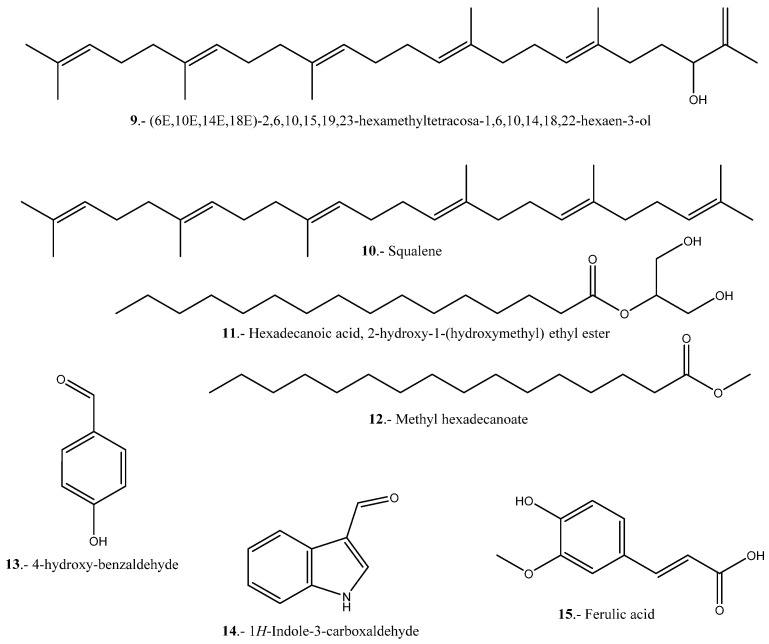
Representative compounds with anticancer activity present in Quinoa and *A. hybridus*.

In a study carried out by Al-Mamun et al. (2016), the anticancer activity of an aqueous extract of the stem of *Amaranthus lividus* (AL) and an aqueous extract of the seeds of *Amaranthus hybridus* (AH) was tested on Ehrlich ascites carcinoma (EAC) cells. The extracts were tested on 42 mature Swiss albino mice, to which the extracts were applied for 6 days. After the administration of the extracts in doses of 25, 50 and 100 µg/mL/d for 6 days, a count of EAC cells was performed with a hemocytometer using trypan blue, whereby it was demonstrated that the AH extract exhibited growth inhibitory activity relatively higher than that of the AL stem extract used in the same concentrations. Therefore, the study shows that Amaranthus exhibits powerful anticancer properties [108]. Antioxidant polysaccharides from *A. hybridus* have been isolated and characterized and shown to possess significant in vitro antioxidant activity associated with cancer prevention [134]. In vitro and in vivo studies on the anticancer activity of both species are shown in Table 8, and notable compounds are shown in Figure 5.

Limitations of the present study are the lack of information about quinoa and amaranth consumption and cancer and other disease rates where the species are traditionally used and the lack of clinical trials to further establish the activity of the studied extracts and compounds.

## 8. Conclusions

*Chenopodium quinoa* and *Amaranthus hybridus* are ancestral pseudocereals that have experienced a revival in the last four decades. Besides their hardiness, which is relevant to attaining food security in the climate crisis we are collectively facing, and their high nutritional content, they possess significant biological activity.

The antimicrobial activity of quinoa is stronger than that of amaranth, which exhibits weak antimicrobial activity as tested. Quinoa exhibits ample antimicrobial properties, including action against antibiotic-resistant strains, due to diverse phytochemical compound classes, mainly phenolics.

Active anticancer compounds are found in both species. Of particular interest are proteins and peptides with anticancer activity, as well as phenolics, unsaponifiable terpenoids and pigments.

The substances that garner the most interest in both species, both for their antibacterial and anticancer activity, are phenolics, peptides and saponins, and there is promising work being carried out on bioactive polysaccharides.

## Figures and Tables

**Table 1:** The SPAR-4-SLR systematic review method followed.

Stage	Substage	
1 Assembling	1a Identification	Domain: Phytochemistry, phytomedicine Research questions: What is the current knowledge about the antimicrobial and anticancer activity of neglected Andean grains *C. quinoa* and *A. hybridus*? Source type: Research articles, reviews and book chapters Source quality: Crossref database
	1b Acquisition	Search mechanism and material acquisition: Dimensions query, ordered by rank Search period: No time constraints Search keywords: (Quinoa OR Amaranth) AND (antimicrobial OR cancer) Total number of articles returned from the search: 221
2 Arranging	2a Organization	Organizing codes: As provided in Dimensions export
	2b Purification	Article type excluded (*n* = 121): Remove duplicates (*n* = 8); remove predatory titles; remove non-empirical, non-review articles. Remove articles not related to the topic or dealing with other *Amaranthus* species Article type included (*n* = 100): Triangulation with previous reviews to ensure seminal articles are included
3 Assessing	3a Evaluation	Analysis method: Content—descriptive Agenda proposal method: Future research directions and identification of existing gaps
	3c Reporting	Reporting conventions: Discussion and summaries in the form of tables and figures Limitations: Discussed Sources of support: Acknowledged

**Table 2:** Exclusion criteria.

Criterion	Excluded Articles
Duplicate article	8
Unrelated topics	112
Corrigenda	1
Total	121

**Table 3:** Example proximal composition of Quinoa and Amaranth grains.

Species	Protein (% dew)	Carbohydrate (% dew)	Fiber (% dw)	Fat (% dw)	Ash (% dw)	Energy (kcal/100 g)
Quinoa	14.1	64.2	7.0	6.1	2.4	353
Amaranth	13.6	65.3	6.7	7.0	6.7	365

Source: [4]. Note: dw: Dry weight.

**Table 4:** Ethnopharmacological uses of *C. quinoa*.

Plant Organ	Mode of Use	Effect	ATC Category	Ref.
Stem Leaves	NS	“Improve the quality of blood”	B	[74]
Leaves	Poultice	Sore throat relief Angina	A C	[49]
Leaves	Decoction	Urinary infections Laxative Rheumatism	G A M	[52]
Leaves (fresh)	Soup or main course	Scurvy and other avitaminoses	A	[49]
Fruit	Poultice or decoction	Wound treatment	D	[49]
Seed	Decoction	Liver abscesses Internal secretions Catarrhal affections	J	[49]
		Bronchial disorders Colds Cough Tonsillitis	R	[49]
	Soaked	Intermittent fevers	J	[52]
	NS	Colon cancer prevention	A	[74]
Seed, stem, leaves	Decoction	Emmenagogue	G	[52]
Leaves	Pounded	Headaches	N	[52]

NS: Not specified. Anatomical Therapeutical Chemical (ATC) categories are as follows. A: Alimentary tract and metabolism; B: Blood and blood-forming organs; C: Cardiovascular system; D: Dermatological; G: Genito-urinary system and sex hormones; J: Anti-infective for systemic use; M: Musculo-skeletal system; N: Nervous system; R: Respiratory system [75].

**Table 5:** Ethnopharmacological uses of *A. hybridus*.

Plant Organ	Mode of Use	Effect	ATC Category	Ref.
All organs	Decoction	Calming Antiacne Heart conditions Antidiarrheal Anti-inflammatory	N D C A	[52,80]
All organs	NS	Carminative	A	[52]
Leaves	Decoction	Cancer prevention	L	[81]
Whole plant	Poultice	Skin conditions Vulnerary	D	[82]
Inflorescence	Decoction	NS	C R G V	[76]

NS: Not specified. Anatomical Therapeutical Chemical (ATC) categories are as follows. A: Alimentary tract and metabolism; C: Cardiovascular system; D: Dermatological; G: Genito-urinary system and sex hormones; L: Antineoplastic and immunomodulating agents; N: Nervous system; R: Respiratory system; V: Various [75]; STDs: Sexually transmitted diseases; Vet: Veterinary.

**Table 6:** Quinoa and Amaranth phytochemical composition.

*C. quinoa*	*A. hybridus*
Phenolic acids Flavonoids Terpenoids Steroids Alkaloids Peptides	Phenolic acids Flavonoids Tannins Steroids Carotenoids

**Table 7:** Antimicrobial activity of compounds found in Quinoa.

No.	Compound	Biological Activity/Model	Effect	Method	Ref.
1	4-hydroxybenzoic acid	*Staphylococcus epidermidis*	IC_50_: 355 µg/mL	Paper disc	[99]
2	Gallic acid	*Pseudomonas aeruginosa*	MIC: 100 µg/mL	Microdilution	[100]
3	Protocatechuic acid	*Yersinia enterocolitica*	MIC: 2.5 mg/mL	Microdilution	[101]
4	*p*-coumaric acid	*Listeria monocytogenes*	IC_50_: 373.4 μM	Spot-test assay	[102]
5	Ferulic acid	*Escherichia coli* *Pseudomonas aeruginosa*	MIC: 100 µg/mL	Microdilution	[100]
6	Kaempferol	*Staphylococcus aureus*	Bacterial film formation inhibition: 64 μg/mL causes 80% inhibition	Crystal violet staining	[103,104]
7	Myricetin	*Escherichia coli*	MIC_50_: 142 μg/mL	Broth microdilution	[105]
8	Oleanolic acid	*Listeria monocytogenes*	MIC_50_: 16 μg/mL	Broth microdilution	[106]

Note: IC_50_: Half-maximal inhibitory concentration; MIC: Minimum inhibitory concentration.

**Table 8:** In vitro and in vivo anticancer studies of *Chenopodium quinoa* Willd and *Amaranthus hybridus* L. extracts and powders.

Plant Organ		Extract	Model			Method	Effect/Mechanism	Ref.
			In Vitro	In Vivo	Biological Model			
*Chenopodium quinoa* Willd								
Colon								
BSO		*n*-hexane	x		HCT116 cells	Hoechst and MTT staining	647.4 µg/mL, apoptosis	[120]
RSO		*n*-hexane	x		HCT116 cells	Hoechst and MTT staining	381.3 µg/mL, apoptosis	[120]
WSO		*n*-hexane	x		HCT116 cells	Hoechst and MTT staining	281.9 µg/mL, apoptosis	[120]
Seed		Ethanol	x		HCT116 cells	MTT	IC_50_ 110.68 µg/mL at 48 h	[121]
Seed		Protein	x		Caco-2	HDAC1	IC_50_ 0.87–1.85 g/L	[135]
Seed		Protein		x	AOM/DSS-induced colorectal cancer in mice	Symptoms/ SCFA production	Symptom mitigation/partially alleviated dysbiosis	[136]
Liver								
Seed		Powder	x		HEPG2	Cell line	IC_50_ 14.6 µg/mL	[122]
Seed		Petroleum ether, ultrasound-assisted extraction	x		SMMC 7721	MTT	121.4 µg/mL (24 h) and 53.4 µg/mL (48 h), inhibition of cell proliferation	[124]
Brain								
Seed		NS	x		U87 Mg	MTT	50 ± 5.0 nM, cytotoxicity	[123]
Seed		NS	x		GBM NULU	MTT	6.6 ± 4.1 nM (24 h), 8.3 ± 1.6 (48 h), 2.3 ± 4.1 (72 h); cytotoxicity	[123]
Seed		NS	x		GBM ZAR	MTT	3.4 ± 1.9 nM (24 h), 7.6 ± 2.7 nM (48 h), 3.3 ± 1.2 nM (72 h); cytotoxicity	[123]
**Breast**								
Seed		Petroleum ether, ultrasound-assisted extraction	x		MCF-7	MTT	83.48 µg/mL (24 h) and 64.67 µg/mL (48 h), inhibition of cell proliferation	[124]
***Amarantus hybridus* L.**								
Leaves		Aqueous		x	Sodium arsenite-induced micronucleated polychromatic erythrocyte in Wistar albino rats	Hematological tests	1 mL of 0.2 g/mL for 14 days, antigenotoxicity	[130]
Stem and leaves		NS	x		Aflatoxin and fumonisin-induced genotoxicity in H4IIE-luc cells	MTT	40 µg/mL, antigenotoxicity	[131]
	Seed	Methanolic		x	Mice treated with EAC cells	Hemocytometer EAC cell count by trypan blue and DAPI staining	25, 50 and 100 µg/mL/d for 6 days; inhibition of cell growth	[108]

Notes: NS: Not specified; BSO: Black quinoa oil; RSO: Red quinoa oil; WSO: White quinoa oil; EAC: Ehrlich ascites carcinoma; HCT 116: Human colon cancer cell line; Caco-2: Human epithelial cancer cells; HEPG2: Human hepatoblastoma cell line; GMB: Primary glioblastoma; H4IIE-luc: Luciferase receptor rat hepatoma cell line; MCF-7: Human breast cancer cells; SMMC 7721: Human liver cancer cells; U87 Mg: Continuous glioblastoma cell line; DAPI: 4′ 6-diamidino-2-phenylindole; DMEM: Dulbecco’s Modified Eagle’s Medium; MTT: 3-(4,5-dimethyl-2-yl)-2,5-diphenyltetrazolium methyl bromide method; TMZ: Temozolomide (chemotherapeutic used in the treatment of glioblastoma); HDAC1: Histone deacetylase 1 inhibitory activity assay; AOM/DSS: Azoxymethane/dextran sulfate sodium; SCFA: Short-chain fatty acids.

## Data Availability

Data sharing is not applicable.

## Supplementary Materials

The following supporting information can be downloaded at: https://www.mdpi.com/article/10.3390/ph16121728/s1, Table S1: Phytochemicals from *Chenopodium quinoa* Willd.; Table S2: Phytochemicals from *Amaranthus hybridus* L.
