# Supplementary material for: Chenopodium quinoa Willd. and Amaranthus hybridus L.: Ancestral Andean Food Security and Modern Anticancer and Antimicrobial Activity
Source: Pharmaceuticals (Basel). 2023 Dec 15;16(12):1728. doi: 10.3390/ph16121728 (PMC10747716; doi:10.3390/ph16121728)
Supplement: Supplementary file 1 [file pharmaceuticals-16-01728-s001.zip › pharmaceuticals-2740444-supplementary.pdf]

Supplementary material for *Chenopodium quinoa* Willd. and *Amaranthus hybridus* L.: Ancestral Andean Food Safety and Modern Anticancer and Anti-microbial Activity

Juan Carlos Romero-Benavides, Evelyn Guaraca-Pino, Rodrigo Duarte-Casar, Marlene Rojas-Le-Fort and Natalia Bailon-Moscoso

Table S1 : Phytochemicals from *Chenopodium quinoa* Willd.

No.	Compound	Id	Is	PO	Biological activity	Identification method	Ref.
				**Benzoic acid analogues**			
1	Benzoic acid	x		Leaves and flour	N.D.	HPLC-DAD	[1,2]
2	4-hydroxybenzoic acid	x		Leaves and seeds	Antimicrobial	HPLC-DAD	[3]
3	2,4 -dihydroxybenzoic acid	x		Seeds	N.D.	HPLC-DAD	[3]
4	2,5 dihydroxybenzoic acid	x		Seeds	N.D.	HPLC-DAD-MS	[4]
5	3,4 dihydroxybenzoic acid	x		Seeds	N.D.	HPLC-DAD	
6	Canthoside	x		Flour	N.D.	HPLC-DAD	[2]
7	*m*- Ethyl digallate	x		Flour	N.D.	HPLC-DAD	
8	Gallic acid	x		Leaves, stems and seeds	Antibacterial Antioxidant	HPLC-DAD	[1,5]
9	1- *O*- Galloyl-β-D-glucose	x		Seeds	N.D.	HPLC-DAD	[2]
10	Protocatechuic acid	x		Stems and seeds	Anticancer Antibacterial Antioxidant	HPLC-DAD	[6,7]
11	Protocatechuic acid 4- *O*-glucoside	x		Flour	Antioxidant	HPLC-DAD	[2]
12	Syringic acid	x		Leaves and seeds	Antioxidant	HPLC DAD	[3,7]
13	Vanillic acid	x		Leaves and seeds	hepatoprotective Anti-inflammatory	HPLC DAD	[3,7,8]
14	Vanillic acid glycosidic ester	x		Seeds	N.D.	HPLC-UV-VIS	[9]
15	Vanillic acid 4- *O*-glucoside	x		Seeds	N.D.	HPLC-DAS-MS	[4]

No.	Compound	Id	Is	PO	Biological activity	Identification method	Ref.
16	Vanillin	x		Seeds	Antioxidant	HPLC-DAS-MS	[2,4,7]
				**Cinnamic acid analogues**			
17	Caffeic acid	x		Seeds	Antioxidant	HPLC-DAD	[3,7]
18	Chlorogenic acid	x		Leaves and seeds	Antidiabetic	HPLC-DAD	[3,10]
19	Cinnamic acid	x		Stems and seeds	N.D.	HPLC-DAD	[11]
20	*o*- coumaric acid	x		Leaves and seeds	Antioxidant	HPLC-DAD	[3,7]
21	*p*- coumaric acid	x		Leaves and seeds	Antimicrobial	HPLC-DAD	[1,12]
22	*p*- coumaric acid glucoside	x		Seeds	N.D.	HPLC-DAS-MS	[4]
23	8,5'- diferulic acid	x		Seeds	N.D.	HPLC and LC-MS	[3]
24	Ferulic acid	x		Leaves, stems and seeds	Antibacterial Anticancer	HPLC-DAD-MS	[3,11,13]
25	Ferulic acid 4- *O*-glucoside	x		Flour	N.D.	HPLC-DAD	[2]
26	Isoferulic acid	x		Seeds	Antioxidant	HPLC-DAD	[4,14]
27	4' geranyloxyferulic acid	x		Seeds	N.D.	HPLC-DAD	[15]
28	Rosmarinic acid	x		Seeds	Antiviral Anti-inflammatory Antioxidant	HPLC-DAD	[3,16,17]
29	Sinapinic acid	x		Leaves and seeds	Antioxidant	HPLC-DAD	[1,18]
				**Flavones**			
30	Acacetin	x		Flour	Anticancer Anti-inflammatory Contraceptive	HPLC-DAD	[2,19,20]
31	Isovitexin	x		Stems and seeds	Antioxidant Anti-inflammatory Antitumor	HPLC-DAD	[11,21]
32	Orientin	x		Seeds	Anticancer(Esmail et al., 2013)	HPLC-DAD	[11,22]
33	Vitexin	x		Stems and seeds	Antithrombotic Anticancer Anti-inflammatory	HPLC-DAD	[11,23,24]
				**Flavonols**			
34	Kaempeferol	x		Leaves and seeds	Antioxidant Antibacterial	HPLC -DAD- MS	[3,25]

No.	Compound	Id	Is	PO	Biological activity	Identification method	Ref.
35	Kaempeferol -3-glucoside	x		Seeds	N.D.	HPLC-DAD-MS	[4,9]
36	Kaempeferol -3-galactoside	x		Seeds	N.D.	HPLC-DAD-MS	[4,9]
37	Kaempeferol 3-*O*-(2,6 di-α-L rhamnopyranosyl) β-D-galactopyranoside	x		Seeds	Antioxidant	HPLC-DAD-MS	[4,9]
38	Kaempeferol 3- *O*- β-D apiofuranosyl (1 → 2)-O-α -L rampyranosyl (1 → 6)- β -D- galactopyran or been	x		Seeds	Antioxidant	HPLC-UV-VIS	[9]
39	Kaempeferol 3- *O*- β-D apiofuranosyl (1 → 2)- β -D-galactopyran or been	x		Seeds	Antioxidant	HPLC-UV-VIS	[9,26]
40	Kaempeferol 3- *O*- α -L rhamnopyranosyl (1 → 2)- β -D-galactopyran or been	x		Seeds	Antioxidant	HPLC-UV-VIS	[26]
41	Kaempeferol 3- *O*- β-D glucuronic acid	x		Seeds	Antioxidant	HPLC-UV-VIS	[9]
42	Kaempeferol 3,7 dirhamnoside	x		Seeds	N.D.	HPLC-DAD-MS	[4]
43	Isoharmentin	x		Leaves	Antitumor Anti-inflammatory Anticoagulant	HPLC-DAD	[11,27–29]
44	Quercetin	x		Leaves and seeds	Cytotoxic Antioxidant	HPLC-DAD-MS	[4,25,30]
45	Quercetin 3- *O*-glucoside	x		Flour	N.D.	HPLC-DAD	[2]
46	Quercetin 3 rutinoside	x		Seeds	N.D.	HPLC-DAD-MS	[4]
47	Quercetin 3 arabinoside	x		Seeds	N.D.	HPLC-DAD-MS	
48	Quercetin 3-O-β-D-apiofuranosyl-(1 → 2) - α -L- rhamnopyranosyl -(1 → 6)- β - D galactopyran or idoarabin or been	x		Seeds	Antioxidant	HPLC-UV-VIS	[26,31]
49	Quercetin 3- *O*- (2,6 di-α-L- rhamnopyranosyl) β-D galactopyranoside	x		Seeds	Antioxidant	HPLC-UV-VIS	[9,26]
50	Quercetin 3- *O* - (2,6 di-*O* - α rhamnopyranosyl) β-glucopyranoside	x		Seeds	N.D.	HPLC-UV-VIS	[31]
51	Quercetin 3- *O*- β -D-apiofuranosyl (1 → 2) O-α -L-rhamnoporanosyl (1 → 6)- β -D- galactopyran	x		Seeds	Antioxidant	HPLC- UV- Vis	[9]

No.	Compound	Id	Is	PO	Biological activity	Identification method	Ref.
	or been 3,45 dimethyl ether						
52	Rutin	x		Leaves, stems and seeds	Antidiabetic Antioxidant	HPLC-DAD	[10,11,32]
53	Morin	x		Stems and seeds	Anti-inflammatory Antitumor	HPLC-DAD-MS	[4,11,26]
54	Myricetin	x		Seeds	Apoptosis Antimicrobial Antihyperglycemic	HPLC	[33,34]
				**Flavanones**			
55	Hesperidin	x		Seeds	Antitumor Antifungal Antioxidant	HPLC-DAD	[11,35,36]
56	Neohesperidin	x		Seeds	Cytotoxic Antifungal Anti-inflammatory	HPLC-DAD	[11,37]
57	Naringin	x		Seeds	Antioxidant Antifungal	HPLC-DAD-MS	[4,38,39]
				**Flavanols**			
58	Catechin	x		Seeds	Antioxidant	HPLC-DAD	[3,40]
59	Epicatechin	x		Seeds	Antioxidant	HPLC-DAD-MS	[4,41]
60	Epigallocatechin	x		Seeds	Antioxidant	HPLC-DAD-MS	[4,42]
				**Isoflavones**			
61	Biochanin A	x		Seeds		HPLC-DAD	[4]
62	Daidzein	x		Seeds	Antioxidant Antithroimbotic	HPLC-UV-VIS	[43–45]
63	Genistein	x		Seeds	Antiproliferative Antioxidant Anti-inflammatory Neuroprotective	HPLC-UV-VIS	[43,46,47]
64	Prunetin	x		Seeds	Anti-inflammatory	HPLC and LC-MS	[3,48]
65	Puerarin	x		Seeds	Anti-inflammatory	HPLC-DAD-MS	[4,49]
				**Monoterpenes**			
66	*cis-* Ascaridol	x		Leaves	N.D.	GC-MS	[50]
67	*cis-* Isoascaridol	x		Leaves	N.D.	GC-MS	
68	Camphene	x		Leaves	N.D.	GC-MS	
69	Camphor	x		Leaves	N.D.	GC-MS	

No.	Compound	Id	Is	PO	Biological activity	Identification method	Ref.
70	*trans*- carveol	x		Leaves	N.D.	GC-MS	
71	*p*- Cimene	x		Leaves	N.D.	GC-MS	
72	*p*- menta 1(7), 8 diene	x		Leaves	N.D.	GC-MS	
73	*trans-p*- menta- 2 in- 1-ol	x		Leaves	N.D.	GC-MS	
74	Pentebioside	x		Flour	N.D.	HPLC-DAD	[2]
75	β- Pinene	x		Leaves	N.D.	GC-MS	
76	Pinocarvone	x		Leaves	N.D.	GC-MS	
77	α-Terpinene	x		Leaves	N.D.	GC-MS	[50]
78	γ-Terpinene	x		Leaves	N.D.	GC-MS	
79	Terpine-1-ol	x		Leaves	N.D.	GC-MS	
80	α-terpinyl acetate	x		Leaves	N.D.	GC-MS	
				**Sesquiterpenes**			
81	Caryophyllene	x		Leaves	N.D.	GC-MS	[50]
				**Triterpenes**			
				Hederagenin and its derivatives			
82	Hederagenin	x		Seeds and bran	Cytotoxic Immunomodulator	GC-MS	[51,52]
83	3- *O*- α-L- Abinopyranosyl hederagenin		x	Seeds	Cytotoxic	HPLC-NMR	[53,54]
84	3- *O*- α-L- Abinopyranosyl hederagenin 28-O-β-D glucopyranosyl ester		x	Flowers, fruits, seeds and bran	N.D.	HPLC-NMR	[55]
85	3- *O* -β-D- glucopyranosyl (1 → 3)- α-L- arabinopyranosyl hederagenin	x		Seeds and bran	Cytotoxic	HPLC-NMR	[56,57]
86	3- *O* -β-D- glucopyranosyl (1 → 3)- β-D-galactopyranosyl hederagenin	x		Saved	N.D.	HPLC-NMR	[56]
87	3- *O* -β-D- glucopyranosyl (1 → 3)- α-L- arabinopyranosyl hederagenin 28-O-β-D glucopyranosyl ester	x		Flowers, fruits, seeds and bran	N.D.	HPLC-NMR	[55]

No.	Compound	Id	Is	PO	Biological activity	Identification method	Ref.
88	3- *O* -β-D-glucopyranosyl (1 → 3)-O-β-D- galactopyranosyl hederagenin 28-O-β-D glucopyranosyl ester	x		Flowers, fruits, seeds and bran	N.D.	HPLC-NMR	
89	3- *O* -β-D-glucopyranosyl (1 → 4)-β-D- glucopyranosyl hederagenin (1 → 4)-β-D-glucopyranosyl hederegenin	x		Seeds	N.D.	HPLC-UV-VIS	[9]
90	3,23 Bis (*O* - β-D-glucopyranosyloxy) acid olean 12- en- 28-oic-28-O-β-D glucopyranosyl (1 → 3)- *α*-L-arabinipyranosyl ester	x		Seeds	N.D.	GC-MS	[58]
91	3- *O* -β-D-glucopyranosyl hederagenin 28-O-β-D glucopyranosyl ester		x	Flowers, fruits, seeds and bran	N.D.	HPLC-NMR	[55]
92	3- *O* -β-D- xylopyranosyl (1 → 3)-β-D-glucopyranosyl hederagenin ester 28-O-β-D glucopyranosyl		x	Saved	N.D.	HPLC-NMR	[56]
				Spergulagenic acid and its derivatives			
93	Spergulagenic acid	x					
94	3- *O* -*α*-L-arabinopyranosyl- (1 → 3)-β-D-acid gluciciranosyl spergulagenic ester-28-O-β-D glucopyranosyl	x		Seeds	N.D.	HPLC-UV-VIS	[59]
95	3- *O* -β-D-glucopyranosyl (1 → 2)-β-D-glucopyranosyl (1 → 3) -*α*-L-arabinopyranosyl spergulagenic acid	x		Saved	N.D.	HPLC-NMR	[56]
96	3- *O* -β-D-glucopyranosyl (1 → 2)-β-D-glucopyranosyl (1 → 3) -*α*-L-arabinopyranosyl acid spergulagenic ester 28-O-β-D glucopyranosyl	x		Seeds	N.D.	HPLC-UV-VIS	[9]

No.	Compound	Id	Is	PO	Biological activity	Identification method	Ref.
				Serjanic acid and its derivatives			
97	Serjanic acid		x	Flowers, fruits, seeds and bran	Cytotoxic	HPLC-NMR	[55,60]
98	3-O-β-D glucopyranosyl ester 3-O- α-L arabinopyranosyl serjanic acid		x	Flowers, fruits, seeds and bran	N.D.	HPLC-NMR	
99	3-O-β-D-glucopyranosyl-(1 → 3)- α -L- acid arabinopyranosyl serjanic 28 -O- β -D glucopyranosyl ester		x	Flowers, fruits, seeds and bran.	N.D.	HPLC-NMR	[55]
100	3-O-β-D- glucopyranosyl (1 → 2)- β-D-glucopyranosyl (1 → 3) - α-L-arabinopyranosyl ester		x	Flowers, fruits, seeds and bran	N.D.	HPLC-NMR	
101	3-O-β-D-glucopyranosyl serjanic 28-O—β-D glucopyranosyl ester		x	Flowers, fruits, seeds and bran	Cytotoxic	HPLC-NMR	
				Phytolacagenic acid and its derivatives			
102	Phytocagenic acid		x	Saved	N.D.	HPLC-NMR	
103	3-O-α-L-arabinopyranosyl photolacagenic acid 28-O- β-D glucopyranosyl ester		x	Flowers, fruits, seeds and bran	N.D.	HPLC-NMR	[56]
104	3-O-α-L-arabinopyranosyl-(1 → 3)- β -D- acid glucopyranosyl phytolactide 28 -O- β -D glucopyranosyl ester	x		Seeds	N.D.	HPLC-UV-VIS	[9]
105	3-O-β-D galactopyranosyl- (1 → 3)- β -D acid glucopyranosyl phytolacagenic 28- O-β-D glucopyranosyl ester		x	Seeds	N.D.	MRI-MS	[26]
106	3-O - β-D glucopyranosyl (1 → 3)- α -L acid arabinopyranosyl phytolactide		x	Seeds	N.D.	HPLC-NMR	[53]

No.	Compound	Id	Is	PO	Biological activity	Identification method	Ref.
107	3-O-β-D glucopyranosyl (1 → 3)- α -L acid arabinopyranosyl phytolactide 28 -O- β -D glucopyranosyl ester	x		Flowers, fruits, seeds and bran	N.D.	HPLC-UV-VIS	
108	3-O-β-D glucopyranosyl (1 → 3)- β -D phytocaginic acid galactopyranoside ester 28-O-β-D glucopyranosyl ester	x		Flowers, fruits, seeds and bran	N.D.	HPLC-UV-VIS	
109	3-O-β-D glucopyranosyl (1 → 2)- β -D glucopyranosyl (1 → 3)- α -L phytocag onic acid arabinopyran or 28-O- β -D glucopyranosyl ester	x		Flowers, fruits, seeds and bran	N.D.	HPLC-UV-VIS	[9]
110	3-O-β-D glucopyranosyl (1 → 4)- β -D glucopyranosyl (1 → 4)- β -D glucopyranosyl acid phytolacagenic 28-O-β-D glucopyranosyl ester	x		Flowers, fruits, seeds and bran	N.D.	HPLC-UV-VIS	
111	3-O-β-D glucopyranosyl (1 → 3)- β -D xylopyranosyl (1 → 2)- β -D glucopyranosyl acid phytocag enic 28-O- β -D glucopyranosyl ester	x		Seeds	N.D.	HPLC-UV-VIS	
				Gypsogenin and its derivatives			
112	Gypsogenin		x	Flowers, fruits, seeds and bran	Cytotoxic	HPLC-NMR	[55,61]
113	3-O-β-D- glucopyranosyl (1 → 3)- α -L arabinopyranosyl 23-oxo-olean-12-en-28-oic ester 28-O-β-D glucopyranosyl		x	Flowers, fruits, seeds and bran	Cytotoxic	HPLC-NMR	
				3- β- hydroxy 27- oxo- olean- 12- en- 28-oic acid and its derivatives			
114	3- β- hydroxy 27- oxo- olean- 12- en- 28-oic acid		x		Cytotoxic	HPLC-NMR	[55]
115	3-O-β-D- glucopyranosyl (1 → 3)- α -L arabinopyranosyl 27-oxo-olean-12-en-28-oic ester 28-O- β -D glucopyranosyl		x	Flowers, fruits, seeds and bran	Cytotoxic	HPLC-NMR	

No.	Compound	Id	Is	PO	Biological activity	Identification method	Ref.
			3- β,23,30 trihydroxy oleane 12- en-c 28- oic acid triterpenoids				
116	3- β,23,30 trihydroxy oleane 12- en-c 28- oic acid		x	Flowers, fruits, seeds and bran	N.D.	HPLC-NMR	[55]
117	3-O-β-D- glucopyranosyl (1 → 3)- α -L arabinopyranosyl acid 3- β,23,30 - trihydroxyoleano- 12-2n- 28-oic 28-O-β-D glucopyranosyl ester		x	Flowers, fruits, seeds and bran	N.D.	HPLC-NMR	
			Oleanolic acid and its derivatives				
118	Oleanolic acid		x	Seeds	Anticancer Anti-inflammatory Antimicrobial Antioxidant	HPLC-NMR	[56,62–65]
119	Methyl oleanate		x	Saved	Anti-inflammatory	HPLC-NMR	[56,66]
120	3-O-α-Arabinopyranosyl (1 → 3) β -D- acid 28-O- β -D- glucopyranoyl ester glucopyranosyl olean olic ester	x		Seeds	N.D.	HPLC-UV-VIS	[59]
121	3- *O*- β-D- glucopyranosyl oleanolic acid		x	Seeds	Antidibaetic	TLC-MRI	[67,68]
122	3-O-β-D glucopyranosyl (1 → 3) α -L acid arabinopyranosyl olean olic ester 28- *O*- β-D glucopyranosyl		x	Flowers, fruits, seeds and bran	N.D.	HPLC-NMR	[55]
123	3-O-β-D glucopyranosyl (1 → 2) β -D glucopyranosyl (1 → 3) α -L acid arabinopyranosyl olean olic 28- *O*- β-D glucopyranosyl ester		x	Flowers, fruits, seeds and bran	N.D.	HPLC-NMR	
124	Oleanolic acid 3- *O*- β -D glucopyranosyl		x	Seeds	N.D.	TLC-MRI	[67]
125	Oleanolic acid 3- *O*- β -D glucopyranosyl 28- *O*- D glucopyranosyl ester		x	Flowers, fruits, seeds and bran	N.D.	HPLC-NMR	[55]
126	3- *O*- D xylopyranosil (1 → 3)- β -D glucopyranosyl oleanolic acid		x	Seeds	N.D.	TLC-MRI	[67]
127	3- *O*- D xylopyranosyl (1 → 3)-6- methyl β -D		x	Seeds	N.D.	TLC-MRI	

No.	Compound	Id	Is	PO	Biological activity	Identification method	Ref.
128	glucopyranosyl oleanolic acid 3- *O*- β-D xylopyranosyl (1 → 3)- β-D-glucopyranosyl acid oleanolic ester 28- *O*- β-D glucopyranosyl		x	Flowers, fruits, seeds and bran	N.D.	HPLC-NMR	[55]
				Tetracyclic triterpenes			
129	Citrostadienol	x		Seeds	Anticomplementary activity	GC-MS	[69,70]
130	Gramisterol	x		Seeds	Anticancer	GC-MS	[69,71]
131	24-methylene cycloartenol	x		Seeds	N.D.	GC-MS	[69]
				Pentacyclic triterpenes			
132	*α*- Amirin	x		Seeds	Antibacterial Antioxidant	GC-MS	[51,72,73]
133	β-Amyrin	x		Seeds	Antibacterial Antioxidant	GC-MS	
134	Erythrodiol	x		Seeds	N.D.	GC-MS	[51]
135	Echinocystic acid	x		Seeds	N.D.	GC-MS	
136	3β, 23 Dihydroxy-oleano-12-en 28,30- dioic acid		x	Seeds	N.D.	HPLC-NMR	[53]
137	2β, 3β, 23 trihydroxy-olean-12-ene-acid 28,30-dioic 30- methyl ester		x	Saved	N.D.	HPLC-NMR	[56]
138	Queretaroic acid	x		Seeds	N.D.	GC-MS	[51]
139	Ursolic acid	x		Seeds	Antispasmodic Contraceptive anticarcinogen	GC-MS	[14,51,74]
				Meroterpenoids			
140	*α*-Tocopherol	x		Seeds	Antioxidant Antihypercholesterolemic Anticancer Neuroprotect	HPLC-DAD	[6,75]
141	β-Tocopherol	x		Seeds	Antioxidant Antihypercholesterolemic Anticancer Neuroprotect	HPLC-DAD	
142	γ-Tocopherol	x		Seeds	Antioxidant Antihypercholesterolemic	HPLC-DAD	
143	δ-Tocopherol	x		Seeds	Antioxidant Antihypercholesterolemic	HPLC-DAD	
144	*α* Tocotrienol	x		Seeds	Antioxidant	HPLC-DAD	[4]

No.	Compound	Id	Is	PO	Biological activity	Identification method	Ref.
					Anti-inflammatory		
145	β Tocotrienol	x		Seeds	Antioxidant Anti-inflammatory	HPLC-DAS	
**Steroids**							
Steroids C_{27}							
146	Cholesterol	x		Seeds		GC-MS	[9]
147	20- Hydroxyecdysone	x		Seeds	Antioxidant	HPLC-UV-VIS	[76,77]
148	20,26-Dihydroxyecdysone	x		Seeds	Antioxidant	HPLC-UV-VIS	[26,77]
149	2-Deoxy-20-hydroxyecdysone		x	Seeds	N.D.	HPLC-NMR	
150	3-epi-2-Deoxy 20-26-hydroxyecdysone		x	Seeds	N.D.	HPLC-NMR	[78]
151	2-Deoxy 20,26 dihydroxyecdysone		x	Seeds	N.D.	HPLC-NMR	
152	22-Hydroxyecdysone 22-Glycolate		x	Seeds	Antioxidant	TLC- NMR	[77,79]
153	24.25 Dehydroinokosterone		x	Seeds	N.D.	HPLC-NMR	[78]
154	25.27 Dehydroinokosterone		x	Seeds	N.D.	HPLC-NMR	
155	Lanosterol	x		Seeds	N.D.	HPLC-MS	[69]
156	Polypodin B		x	Seeds	N.D.	HPLC-NMR	[78]
Steroids C_{28}							
157	Brassicasterol	x		Seeds	N.D.	HPLC-MS	[80]
158	Campestanol	x		Seeds	N.D.	HPLC-MS	
159	Campesterol	x		Seeds	N.D.	HPLC-MS	[69]
160	Δ^7 Campesterol	x		Seeds	N.D.	HPLC-MS	
161	Dacristerone		x	Seeds	N.D.	HPLC-NMR	[78]
162	Episterol	x		Seeds	N.D.	HPLC-MS	[69]
163	Ergost-7-en-3-ol	x		Seeds	N.D.	HPLC-MS	[69]
164	Cancholosterone	x		Seeds	N.D.	HPLC-UV-VIS	[9]
165	Makisterone A	x		Seeds	Antioxidant	HPLC-UV-VIS	
166	24- *epi*- Makisterone A	x		Seeds	Antioxidant	HPLC-UV-VIS	[26,77]
167	24-Methyl-20,26 Dihydroxyecdysone		x	Seeds	Antioxidant	HPLC-NMR	

No.	Compound	Id	Is	PO	Biological activity	Identification method	Ref.
168	24(28) Dehydromakisterone A	x		Seeds	Antioxidant	HPLC-UV-VIS	
169	26-Hydroxy 24(28) dehygromakisterone		x	Seeds	N.D.	TLC- NMR	[79]
170	5-$^{\beta}$ Hydroxy 24(28) dehydromakisterone A	x		Seeds	N.D.	HPLC-NMR	[78]
				Steroids C_{29}			
171	Δ^{5} Avenasterol	x		Seeds	N.D.	GC-MS	[81]
172	Δ^{7} Avenasterol	x		Seeds	N.D.	HPLC-MS	
173	Makisterone C	x		Seeds	N.D.	HPLC-MS	[69]
174	Sitostanol	x		Seeds	N.D.	HPLC-MS	
175	β Sitosterol	x		Seeds	Antioxidant Antimutagenic Apopototic	GC-MS	[81,82]
176	Δ^{7} Stigmastenol	x		Seeds	Antitumor Cytotoxic Antihypercholesterolemic	HPLC-MS	[69,83,84]
177	Δ^{7} Stigmasterol	x		Seeds	N.D.	GC-MS	[81]
				Nitrogen-containing metabolites			
178	Amaranthine	x		Seeds	N.D.	HPLC-PDA	[85]
179	Betanin	x		Seeds	Antioxidant	HPLC-DAD	[4,86]
180	Isobetanine	x		Seeds	N.D.	HPLC-DAD	[4]
181	Betaine	x		Seeds	N.D.	HPLC-DAD	[4]
182	3-Carboxy-1(2-sulfoethyl)pyridine	x		Seeds	N.D.	HPLC-DAD	
183	Dopaxanthin	x		Seeds	N.D.	HPLC-PDA	
184	Indicaxanthin	x		Seeds	N.D.	HPLC-PDA	[85]
185	Miraxanthin V	x		Seeds	N.D.	HPLC-PDA	
186	Trigonelline	x		Seeds	Antiinvasive activity in cell lines Hypoglycemic	HPLC-UV-VIS	[9,87,88]
187	Trigoneline glucosyl ester	x		Seeds	N.D.	HPLC-UV-VIS	[9]
188	Trigoneline methyl ester	x		Seeds	N.D.	HPLC-UV-VIS	

Note: Is: Isolated; DAD: Diode Array Detector; PO: Plant organ; GC: Gas chromatography; HPLC: High Performance Liquid Chromatography; Id: Identified; MS: Mass spectrometry; ND: Not determined; PDA: Photodiode

array detector; NMR: Nuclear magnetic resonance; TLC: Thin layer chromatography; UV: Ultraviolet; VIS: Visible.

Table S2 : Phytochemicals from *Amaranthus hybridus* L.

No.	Compound	Id	Is	PO	Bioactivity	Method	Refs
	**Hydroxybenzoic acid analogues**						
1	Ellagic acid	x		Leaves	Antioxidant<br>Anticancer<br>Anti-inflammatory	HPLC-DAD	[89–92]
2	Gallic acid	x		Leaves	Antibacterial<br>Antioxidant	HPLC-DAD	[5,89]
	**Hydroxycinnamic acid analogues**						
3	Caffeic acid	x		Leaves	Antioxidant	HPLC-DAD	[7,89]
4	Citric acid-2-caffeoyl	x		Leaves	N.D.	HPLC-DAD	[93]
5	Chlorogenic acid	x		Leaves	Antidiabetic	HPLC-DAD	[10,89]
	**Flavonols**						
6	Kaempferol	x		Dried and cooked leaves	Antioxidant<br>Antibacterial	HPLC-DAD	[3,94]
7	Myricetin	x		Dried and cooked leaves	Apoptosis of breast cancer cells<br>Antihyperglycemic<br>Antibacterial	HPLC-DAD	[33,34,94]
8	Quercetin	x		Leaves, flowers, stems	Cytotoxic in the A549 cell line<br>Antioxidant	HPLC-DAD	[30,94,95]
9	Rutin	x		Leaves<br>Seeds	Antioxidant<br>Antidiabetic	HPLC-DAD	[10,32,89,96]
	**Flavones**						
10	Vitexin	x		Leaves	Anticancer<br>Antioxidant	HPLC-DAD	[23,24,89]
12	Apigenin	x		Leaves	Breast cancer<br>Anti-inflammatory	HPLC-DAD	[89,97]
	**Carotenoids**						
13	β-carotene	x		Raw and boiled leaves	Antioxidant	HPLC-DAD	[98,99]
14	Lutein	x		Raw and boiled leaves	Antioxidant	HPLC-DAD	[98,100]
	**Nitrogen-containing compounds**						
15	Amaranthine	x		Seeds	Antioxidant	HPLC-MS	[101,102]
16	Isoananthine	x		Seeds	Antioxidant	HPLC-MS	

Note: Is: Isolated; DAD: Diode Array Detector; PO: Plant organ; GC: Gas chromatography; HPLC: High Performance Liquid Chromatography; Id: Identified; MS: Mass spectrometry; ND: Not determined; PDA: Photodiode array detector; NMR: Nuclear magnetic resonance; TLC: Thin layer chromatography; UV: Ultraviolet; VIS: Visible.

References

1. Gawlik-Dziki, U.; Świeca, M.; Sułkowski, M.; Dziki, D.; Baraniak, B.; Czyż, J. Antioxidant and Anticancer Activities of Chenopodium Quinoa Leaves Extracts – In Vitro Study. *Food Chem. Toxicol.* **2013**, *57*, 154–160, doi:10.1016/j.fct.2013.03.023.
2. Gómez-Caravaca, A.M.; Segura-Carretero, A.; Fernández-Gutiérrez, A.; Caboni, M.F. Simultaneous Determination of Phenolic Compounds and Saponins in Quinoa (Chenopodium Quinoa Willd) by a Liquid Chromatography–Diode Array Detection–Electrospray Ionization–Time-of-Flight Mass Spectrometry Methodology. *J. Agric. Food Chem.* **2011**, *59*, 10815–10825, doi:10.1021/jf202224j.
3. Tang, Y.; Zhang, B.; Li, X.; Chen, P.X.; Zhang, H.; Liu, R.; Tsao, R. Bound Phenolics of Quinoa Seeds Released by Acid, Alkaline, and Enzymatic Treatments and Their Antioxidant and α-Glucosidase and Pancreatic Lipase Inhibitory Effects. *J. Agric. Food Chem.* **2016**, *64*, 1712–1719, doi:10.1021/acs.jafc.5b05761.
4. Tang, Y.; Li, X.; Zhang, B.; Chen, P.X.; Liu, R.; Tsao, R. Characterisation of Phenolics, Betanins and Antioxidant Activities in Seeds of Three Chenopodium Quinoa Willd. Genotypes. *Food Chem.* **2015**, *166*, 380–388, doi:10.1016/j.foodchem.2014.06.018.
5. Qanash, H.; Yahya, R.; Bakri, M.M.; Bazaid, A.S.; Qanash, S.; Shater, A.F.; T. M., A. Anticancer, Antioxidant, Antiviral and Antimicrobial Activities of Kei Apple (Dovyalis Caffra) Fruit. *Sci. Rep.* **2022**, *12*, 5914, doi:10.1038/s41598-022-09993-1.
6. Alvarez-Jubete, L.; Wijngaard, H.; Arendt, E.K.; Gallagher, E. Polyphenol Composition and in Vitro Antioxidant Activity of Amaranth, Quinoa Buckwheat and Wheat as Affected by Sprouting and Baking. *Food Chem.* **2010**, *119*, 770–778, doi:10.1016/j.foodchem.2009.07.032.
7. Ti, H.; Li, Q.; Zhang, R.; Zhang, M.; Deng, Y.; Wei, Z.; Chi, J.; Zhang, Y. Free and Bound Phenolic Profiles and Antioxidant Activity of Milled Fractions of Different Indica Rice Varieties Cultivated in Southern China. *Food Chem.* **2014**, *159*, 166–174, doi:10.1016/j.foodchem.2014.03.029.
8. Itoh, A.; Isoda, K.; Kondoh, M.; Kawase, M.; Kobayashi, M.; Tamesada, M.; Yagi, K. Hepatoprotective Effect of Syringic Acid and Vanillic Acid on Concanavalin A-Induced Liver Injury. *Biol. Pharm. Bull.* **2009**, *32*, 1215–1219, doi:10.1248/bpb.32.1215.
9. Dini, I.; Carlo Tenore, G.; Dini, A. Phenolic Constituents of Kancolla Seeds. *Food Chem.* **2004**, *84*, 163–168, doi:10.1016/S0308-8146(03)00185-7.
10. Hunyadi, A.; Martins, A.; Hsieh, T.-J.; Seres, A.; Zupkó, I. Chlorogenic Acid and Rutin Play a Major Role in the In Vivo Anti-Diabetic Activity of Morus Alba Leaf Extract on Type II Diabetic Rats. *PLOS ONE* **2012**, *7*, e50619, doi:10.1371/journal.pone.0050619.
11. Paśko, P.; Sajewicz, M.; Gorinstein, S.; Zachwieja, Z. Analysis of Selected Phenolic Acids and Flavonoids in Amaranthus Cruentus and Chenopodium Quinoa Seeds and Sprouts by HPLC. *Acta Chromatogr.* **2008**, *20*, 661–672, doi:10.1556/achrom.20.2008.4.11.
12. Wen, A.; Delaquis, P.; Stanich, K.; Toivonen, P. Antilisterial Activity of Selected Phenolic Acids. *Food Microbiol.* **2003**, *20*, 305–311, doi:10.1016/S0740-0020(02)00135-1.
13. Tsou, M.F.; Hung, C.F.; Lu, H.F.; Wu, L.T.; Chang, S.H.; Chang, H.L.; Chen, G.W.; Chung, J.G. Effects of Caffeic Acid, Chlorogenic Acid and Ferulic Acid on Growth and Arylamine N-Acetyltransferase Activity in Shigella Sonnei (Group D). *Microbios* **2000**, *101*, 37–46.
14. Wang, C.-M.; Yeh, K.-L.; Tsai, S.-J.; Jhan, Y.-L.; Chou, C.-H. Anti-Proliferative Activity of Triterpenoids and Sterols Isolated from Alstonia Scholaris against Non-Small-Cell Lung Carcinoma Cells. *Mol. Basel Switz.* **2017**, *22*, 2119, doi:10.3390/molecules22122119.
15. Fiorito, S.; Preziuso, F.; Epifano, F.; Scotti, L.; Bucciarelli, T.; Taddeo, V.A.; Genovese, S. Novel Biologically Active Principles from Spinach, Goji and Quinoa. *Food Chem.* **2019**, *276*, 262–265, doi:10.1016/j.foodchem.2018.10.018.
16. Cao, H.; Cheng, W.-X.; Li, C.; Pan, X.-L.; Xie, X.-G.; Li, T.-H. DFT Study on the Antioxidant Activity of Rosmarinic Acid. *J. Mol. Struct. THEOCHEM* **2005**, *719*, 177–183, doi:10.1016/j.theochem.2005.01.029.

17. Swarup, V.; Ghosh, J.; Ghosh, S.; Saxena, A.; Basu, A. Antiviral and Anti-Inflammatory Effects of Rosmarinic Acid in an Experimental Murine Model of Japanese Encephalitis. *Antimicrob. Agents Chemother.* **2007**, *51*, 3367–3370, doi:10.1128/aac.00041-07.
18. Galano, A.; Francisco-Márquez, M.; Alvarez-Idaboy, J.R. Mechanism and Kinetics Studies on the Antioxidant Activity of Sinapinic Acid. *Phys. Chem. Chem. Phys.* **2011**, *13*, 11199–11205, doi:10.1039/C1CP20722A.
19. Liu, L.-Z.; Jing, Y.; Jiang, L.L.; Jiang, X.-E.; Jiang, Y.; Rojanasakul, Y.; Jiang, B.-H. Acacetin Inhibits VEGF Expression, Tumor Angiogenesis and Growth through AKT/HIF-1α Pathway. *Biochem. Biophys. Res. Commun.* **2011**, *413*, 299–305, doi:10.1016/j.bbrc.2011.08.091.
20. Semwal, R.B.; Semwal, D.K.; Combrinck, S.; Trill, J.; Gibbons, S.; Viljoen, A. Acacetin—A Simple Flavone Exhibiting Diverse Pharmacological Activities. *Phytochem. Lett.* **2019**, *32*, 56–65, doi:10.1016/j.phytol.2019.04.021.
21. Carballo-Villalobos, A.I.; González-Trujano, M.I.; López-Muñoz, F.J. Evidence of Mechanism of Action of Anti-Inflammatory/Antinociceptive Activities of Acacetin. *Eur. J. Pain* **2014**, *18*, 396–405, doi:10.1002/j.1532-2149.2013.00378.x.
22. Kim, S.-J.; Pham, T.-H.; Bak, Y.; Ryu, H.-W.; Oh, S.-R.; Yoon, D.-Y. Orientin Inhibits Invasion by Suppressing MMP-9 and IL-8 Expression via the PKCα/ ERK/AP-1/STAT3-Mediated Signaling Pathways in TPA-Treated MCF-7 Breast Cancer Cells. *Phytomedicine* **2018**, *50*, 35–42, doi:10.1016/j.phymed.2018.09.172.
23. Abbasi, E.; Nassiri-Asl, M.; Sheikhi, M.; Shafiee, M. Effects of Vitexin on Scopolamine-Induced Memory Impairment in Rats. *Chin. J. Physiol.* **2013**, *56*, 184–189, doi:10.4077/CJP.2013.BAB123.
24. Borghi, S.M.; Carvalho, T.T.; Staurengo-Ferrari, L.; Hohmann, M.S.N.; Pinge-Filho, P.; Casagrande, R.; Verri, W.A.Jr. Vitexin Inhibits Inflammatory Pain in Mice by Targeting TRPV1, Oxidative Stress, and Cytokines. *J. Nat. Prod.* **2013**, *76*, 1141–1149, doi:10.1021/np400222v.
25. Jan, R.; Khan, M.; Asaf, S.; Lubna; Asif, S.; Kim, K.-M. Bioactivity and Therapeutic Potential of Kaempferol and Quercetin: New Insights for Plant and Human Health. *Plants* **2022**, *11*, 2623, doi:10.3390/plants11192623.
26. ZHU, N.; SHENG, S.; LI, D.; LAVOIE, E.J.; KARWE, M.V.; ROSEN, R.T.; HO, C. Antioxidative Flavonoid Glycosides from Quinoa Seeds (*Chenopodium Quinoa* Willd.). *J. Food Lipids* **2001**, *8*, 37–44, doi:10.1111/j.1745-4522.2001.tb00182.x.
27. Li, C.; Yang, D.; Zhao, Y.; Qiu, Y.; Cao, X.; Yu, Y.; Guo, H.; Gu, X.; Yin, X. Inhibitory Effects of Isorhamnetin on the Invasion of Human Breast Carcinoma Cells by Downregulating the Expression and Activity of Matrix Metalloproteinase-2/9. *Nutr. Cancer* **2015**, *67*, 1191–1200, doi:10.1080/01635581.2015.1073763.
28. Chirumbolo, S. Anti-Inflammatory Action of Isorhamnetin. *Inflammation* **2014**, *37*, 1200–1201, doi:10.1007/s10753-014-9846-9.
29. Ku, S.-K.; Kim, T.H.; Bae, J.-S. Anticoagulant Activities of Persicarin and Isorhamnetin. *Vascul. Pharmacol.* **2013**, *58*, 272–279, doi:10.1016/j.vph.2013.01.005.
30. Pereira, E.; Cadavez, V.; Barros, L.; Encina-Zelada, C.; Stojković, D.; Sokovic, M.; Calhelha, R.C.; Gonzales-Barron, U.; Ferreira, I.C.F.R. Chenopodium Quinoa Willd. (Quinoa) Grains: A Good Source of Phenolic Compounds. *Food Res. Int.* **2020**, *137*, 109574, doi:10.1016/j.foodres.2020.109574.
31. Hirose, Y.; Fujita, T.; Ishii, T.; Ueno, N. Antioxidative Properties and Flavonoid Composition of Chenopodium Quinoa Seeds Cultivated in Japan. *Food Chem.* **2010**, *119*, 1300–1306, doi:10.1016/j.foodchem.2009.09.008.
32. Ng, T.B.; Liu, F.; Wang, Z.T. Antioxidative Activity of Natural Products from Plants. *Life Sci.* **2000**, *66*, 709–723, doi:10.1016/S0024-3205(99)00642-6.
33. Han, S.-H.; Lee, J.-H.; Woo, J.-S.; Jung, G.-H.; Jung, S.-H.; Han, E.-J.; Kim, B.; Cho, S.D.; Nam, J.S.; Che, J.H.; et al. Myricetin Induces Apoptosis and Autophagy in Human Gastric Cancer

Cells through Inhibition of the PI3K/Akt/mTOR Pathway. *Heliyon* **2022**, *8*, doi:10.1016/j.heliyon.2022.e09309.

34. Devnath, H.S.; Ahmed, Md.I.; Medha, M.M.; Islam, Md.N.; Biswas, R.P.; Islam, Md.A.; Sadhu, S.K. HPLC Analysis and Antimicrobial, Antidiarrheal and Antihyperglycemic Properties of Eurya Acuminata along with in Silico Profiles. *Phytomedicine Plus* **2022**, *2*, 100291, doi:10.1016/j.phyplu.2022.100291.
35. Saleh, N.; Allam, T.; Korany, R.M.S.; Abdelfattah, A.M.; Omran, A.M.; Eldaim, M.A.A.; Hassan, A.M.; El-Borai, N.B. Protective and Therapeutic Efficacy of Hesperidin versus Cisplatin against Ehrlich Ascites Carcinoma-Induced Renal Damage in Mice. *Pharmaceuticals* **2022**, *15*, 294, doi:10.3390/ph15030294.
36. Al-Ashaal, H.A.; El-Sheltawy, S.T. Antioxidant Capacity of Hesperidin from Citrus Peel Using Electron Spin Resonance and Cytotoxic Activity against Human Carcinoma Cell Lines. *Pharm. Biol.* **2011**, *49*, 276–282, doi:10.3109/13880209.2010.509734.
37. Ortiz, A. de C.; Fideles, S.O.M.; Reis, C.H.B.; Bellini, M.Z.; Pereira, E. de S.B.M.; Pilon, J.P.G.; de Marchi, M.Â.; Detregiachi, C.R.P.; Flato, U.A.P.; Trazzi, B.F. de M.; et al. Therapeutic Effects of Citrus Flavonoids Neohesperidin, Hesperidin and Its Aglycone, Hesperetin on Bone Health. *Biomolecules* **2022**, *12*, 626, doi:10.3390/biom12050626.
38. Nasrollahi, H.; Karimi, M.; Raofie, F. Comparison of Supercritical Fluid Extraction and Ultrasound-Assisted Extraction of Corchorus Olitorius L. and Evaluation of Their Antioxidant Properties and Identification of Compounds Using LC-ESI-MS. *Tradit. Integr. Med.* **2022**, doi:10.18502/tim.v7i1.9065.
39. Pok, P.S.; Londoño, V.A.G.; Vicente, S.; Pacin, A.; Alzamora, S.M.; Resnik, S.L. Citrus Flavonoids against Fusarium Verticillioides in Post-harvest Maize: Minimization of Fumonisins and Alteration of Fungal Ultrastructure. *J. Appl. Microbiol.* **2022**, *132*, 2234–2248, doi:10.1111/jam.15373.
40. Hirasawa, M.; Takada, K. Multiple Effects of Green Tea Catechin on the Antifungal Activity of Antimycotics against Candida Albicans. *J. Antimicrob. Chemother.* **2004**, *53*, 225–229, doi:10.1093/jac/dkh046.
41. Iacopini, P.; Baldi, M.; Storchi, P.; Sebastiani, L. Catechin, Epicatechin, Quercetin, Rutin and Resveratrol in Red Grape: Content, in Vitro Antioxidant Activity and Interactions. *J. Food Compos. Anal.* **2008**, *21*, 589–598, doi:10.1016/j.jfca.2008.03.011.
42. Komolafe, K.; Olaleye, T.M.; Omotuyi, O.I.; Boligon, A.A.; Athayde, M.L.; Akindahunsi, A.A.; da Rocha, J.B.T. In Vitro Antioxidant Activity and Effect of Parkia Biglobosa Bark Extract on Mitochondrial Redox Status. *J. Acupunct. Meridian Stud.* **2013**, *7*, 202–210, doi:10.1016/j.jams.2013.08.003.
43. Lutz, M.; Martínez, A.; Martínez, E.A. Daidzein and Genistein Contents in Seeds of Quinoa (Chenopodium Quinoa Willd.) from Local Ecotypes Grown in Arid Chile. *Ind. Crops Prod.* **2013**, *49*, 117–121, doi:10.1016/j.indcrop.2013.04.023.
44. Foti, P.; Erba, D.; Riso, P.; Spadafranca, A.; Criscuoli, F.; Testolin, G. Comparison between Daidzein and Genistein Antioxidant Activity in Primary and Cancer Lymphocytes. *Arch. Biochem. Biophys.* **2005**, *433*, 421–427, doi:10.1016/j.abb.2004.10.008.
45. Choo, M.-K.; Park, E.-K.; Yoon, H.-K.; Kim, D.-H. Antithrombotic and Antiallergic Activities of Daidzein, a Metabolite of Puerarin and Daidzin Produced by Human Intestinal Microflora. *Biol. Pharm. Bull.* **2002**, *25*, 1328, doi:10.1248/bpb.25.1328.
46. CHOI, E.J.; KIM, G.-H. Antiproliferative Activity of Daidzein and Genistein May Be Related to ERα/c-erbB-2 Expression in Human Breast Cancer Cells. *Mol. Med. Rep.* **2013**, *7*, 781–784, doi:10.3892/mmr.2013.1283.
47. Aldhahri, R.S.; Alghamdi, B.S.; Alzahrani, N.A.; Bahaidrah, K.A.; Alsufiani, H.M.; Mansouri, R.A.; Ashraf, G.M. Biochanin A Improves Memory Decline and Brain Pathology in Cuprizone-Induced Mouse Model of Multiple Sclerosis. *Behav. Sci.* **2022**, *12*, 70, doi:10.3390/bs12030070.

48. Yang, G.; Ham, I.; Choi, H.-Y. Anti-Inflammatory Effect of Prunetin via the Suppression of NF-κB Pathway. *Food Chem. Toxicol.* **2013**, *58*, 124–132, doi:10.1016/j.fct.2013.03.039.
49. Song, X.; Wang, W.; Ding, S.; Wang, Y.; Ye, L.; Chen, X.; Ma, H. Exploring the Potential Antidepressant Mechanisms of Puerarin: Anti-Inflammatory Response via the Gut-Brain Axis. *J. Affect. Disord.* **2022**, *310*, 459–471, doi:10.1016/j.jad.2022.05.044.
50. Dembitsky, V.; Shkrob, I.; Hanus, L.O. Ascaridole and Related Peroxides from the Genus Chenopodium. *Biomed. Pap.* **2008**, *152*, 209–215, doi:10.5507/bp.2008.032.
51. Burnouf-Radosevich, M.; Delfel, N.E.; England, R. Gas Chromatography-Mass Spectrometry of Oleanane- and Ursane-Type Triterpenes—Application to Chenopodium Quinoa Triterpenes. *Phytochemistry* **1985**, *24*, 2063–2066, doi:10.1016/s0031-9422(00)83122-2.
52. Sarikahya, N.B.; Mkouboi, M.C.; Nalbantsoy, A.; Elibol, M. Cytotoxic and Immunomodulator Potential of Hederagenin Saponins from Cephalaria Tchihatchewii. *Phytochem. Lett.* **2021**, *44*, 216–221, doi:10.1016/j.phytol.2020.12.007.
53. Woldemichael, G.M.; Wink, M. Identification and Biological Activities of Triterpenoid Saponins from Chenopodium Quinoa. *J. Agric. Food Chem.* **2001**, *49*, 2327–2332, doi:10.1021/jf0013499.
54. Barthomeuf, C.; Debiton, E.; Mshvildadze, V.; Kemertelidze, E.; Balansard, G. In Vitro Activity of Hederacolchisid A1 Compared with Other Saponins from Hedera Colchica Against Proliferation of Human Carcinoma and Melanoma Cells. *Planta Med.* **2002**, *68*, 672–675, doi:10.1055/s-2002-33807.
55. Kuljanabhagavad, T.; Thongphasuk, P.; Chamulitrat, W.; Wink, M. Triterpene Saponins from Chenopodium Quinoa Willd. *Phytochemistry* **2008**, *69*, 1919–1926, doi:10.1016/j.phytochem.2008.03.001.
56. Mizui, F.; Kasai, R.; Ohtani, K.; Tanaka, O. Saponins from Brans of Quinoa, Chenopodium Quinoa WILLD. I. *Chem. Pharm. Bull. (Tokyo)* **1988**, *36*, 1415–1418, doi:10.1248/cpb.36.1415.
57. Jung, H.-J.; Lee, C.O.; Lee, K.-T.; Choi, J.; Park, H.-J. Structure–Activity Relationship of Oleanane Disaccharides Isolated from Akebia Quinata versus Cytotoxicity against Cancer Cells and NO Inhibition. *Biol. Pharm. Bull.* **2004**, *27*, 744, doi:10.1248/bpb.27.744.
58. Meyer, B.N.; Heinstein, P.F.; Burnouf-Radosevich, M.; Delfel, N.E.; McLaughlin, J.L. Bioactivity-Directed Isolation and Characterization of Quinoside A: One of the Toxic/Bitter Principles of Quinoa Seeds (Chenopodium Quinoa Willd.). *J. Agric. Food Chem.* **1990**, *38*, 205–208, doi:10.1021/jf00091a045.
59. Dini, I.; Tenore, G.C.; Schettino, O.; Dini, A. New Oleanane Saponins in Chenopodium Quinoa. *J. Agric. Food Chem.* **2001**, *49*, 3976–3981, doi:10.1021/jf010361d.
60. El Hazzam, K.; Hafsa, J.; Sobeh, M.; Mhada, M.; Taourirte, M.; EL Kacimi, K.; Yasri, A. An Insight into Saponins from Quinoa (Chenopodium Quinoa Willd): A Review. *Molecules* **2020**, *25*, 1059, doi:10.3390/molecules25051059.
61. Kraft, O.; Hartmann, A.-K.; Hoenke, S.; Serbian, I.; Csuk, R. Madecassic Acid—A New Scaffold for Highly Cytotoxic Agents. *Int. J. Mol. Sci.* **2022**, *23*, 4362, doi:10.3390/ijms23084362.
62. Dzubak, P.; Hajduch, M.; Vydra, D.; Hustova, A.; Kvasnica, M.; Biedermann, D.; Markova, L.; Urban, M.; Sarek, J. Pharmacological Activities of Natural Triterpenoids and Their Therapeutic Implications. *Nat. Prod. Rep.* **2006**, *23*, 394–411, doi:10.1039/b515312n.
63. Zhang, Z.-S.; Zeng, Q.-Y.; Liu, Y.-J. Frequent Ploidy Changes in Salicaceae Indicates Widespread Sharing of the Salicoid Whole Genome Duplication by the Relatives of Populus L. and Salix L. *BMC Plant Biol.* **2021**, *21*, 535, doi:10.1186/s12870-021-03313-x.
64. Wolska, K.; Grudniak, A.; Fiecek, B.; Kraczkiewicz-Dowjat, A.; Kurek, A. Antibacterial Activity of Oleanolic and Ursolic Acids and Their Derivatives. *Open Life Sci.* **2010**, *5*, 543–553, doi:10.2478/s11535-010-0045-x.
65. Wang, N.; Hatcher, D.W.; Tyler, R.T.; Toews, R.; Gawalko, E.J. Effect of Cooking on the Composition of Beans (Phaseolus Vulgaris L.) and Chickpeas (Cicer Arietinum L.). *Food Res. Int.* **2010**, *43*, 589–594, doi:10.1016/j.foodres.2009.07.012.

66. Pérez-Lozano, J. Entrevista a Philippe Regol, Premio Nacional de Gastronomía. *Gastroactitud* 2013.
67. Ma, W.W.; Heinstein, P.F.; McLaughlin, J.L. Additional Toxic, Bitter Saponins from the Seeds of Chenopodium Quinoa. *J. Nat. Prod.* **1989**, *52*, 1132–1135, doi:10.1021/np50065a035.
68. Yoshikawa, M.; Matsuda, H. Antidiabetogenic Activity of Oleanolic Acid Glycosides from Medicinal Foodstuffs. *BioFactors Oxf. Engl.* **2000**, *13*, 231–237, doi:10.1002/biof.5520130136.
69. Fanali, C.; Beccaria, M.; Salivo, S.; Tranchida, P.; Tripodo, G.; Farnetti, S.; Dugo, L.; Dugo, P.; Mondello, L. Non-Polar Lipids Characterization of Quinoa (Chenopodium Quinoa) Seed by Comprehensive Two-Dimensional Gas Chromatography with Flame Ionization/Mass Spectrometry Detection and Non-Aqueous Reversed-Phase Liquid Chromatography with Atmospheric Pressure Chemical Ionization Mass Spectrometry Detection. *J. Sep. Sci.* **2015**, *38*, 3151–3160, doi:10.1002/jssc.201500466.
70. Lee, I.S.; Oh, S.R.; Jung, K.Y.; Kim, D.S.; Kim, J.H.; Lee, H.-K. Anticomplementary Activity and Complete 13C NMR Assignment of Citrostadienol from *Schizandra Chinensis*. *Int. J. Pharmacogn.* **1997**, *35*, 358–363, doi:10.1080/09251619708951283.
71. Suttiarporn, P.; Chumpolsri, W.; Mahatheeranont, S.; Luangkamin, S.; Teepsawang, S.; Leardkamolkarn, V. Structures of Phytosterols and Triterpenoids with Potential Anti-Cancer Activity in Bran of Black Non-Glutinous Rice. *Nutrients* **2015**, *7*, 1672–1687, doi:10.3390/nu7031672.
72. Saeed, M.A.; Sabir, A.W. Antibacterial Activity of Caesalpinia Bonducella Seeds. *Fitoterapia* **2001**, *72*, 807–809, doi:10.1016/s0367-326x(01)00292-1.
73. Cardoso, B.K.; de Oliveira, H.L.M.; Melo, U.Z.; Fernandez, C.M.M.; de Araújo Almeida Campo, C.F.; Gonçalves, J.E.; Laverde, A.; Romagnolo, M.B.; Linde, A.; Gazim, Z.C. Antioxidant Activity of α and β-Amyrin Isolated from Myrcianthes Pungens Leaves. *Nat. Prod. Res.* **2018**, *34*, 1777–1781, doi:10.1080/14786419.2018.1525715.
74. González-Trujano, M.; Ventura-Martínez, R.; Chávez, M.; Díaz-Reval, I.; Pellicer, F. Spasmolytic and Antinociceptive Activities of Ursolic Acid and Acacetin Identified in Agastache Mexicana. *Planta Med.* **2012**, *78*, 793–796, doi:10.1055/s-0031-1298416.
75. Sookwong, P.; Murata, K.; Nakagawa, K.; Shibata, A.; Kimura, T.; Yamaguchi, M.; Kojima, Y.; Miyazawa, T. Cross-Fertilization for Enhancing Tocotrienol Biosynthesis in Rice Plants and QTL Analysis of Their F2 Progenies. *J. Agric. Food Chem.* **2009**, *57*, 4620–4625, doi:10.1021/jf900394t.
76. Muchate, N.S.; Rajurkar, N.S.; Suprasanna, P.; Nikam, T.D. Evaluation of Spinacia Oleracea (L.) for Phytodesalination and Augmented Production of Bioactive Metabolite, 20-Hydroxyecdysone. *Int. J. Phytoremediation* **2018**, *20*, 981–994, doi:10.1080/15226514.2018.1452184.
77. Zhu, N.; Kikuzaki, H.; Vastano, B.C.; Nakatani, N.; Karwe, M.V.; Rosen, R.T.; Ho, C.-T. Ecdysteroids of Quinoa Seeds (Chenopodium Quinoa Willd.). *J. Agric. Food Chem.* **2001**, *49*, 2576–2578, doi:10.1021/jf0014462.
78. Kumpun, S.; Maria, A.; Crouzet, S.; Evrard-Todeschi, N.; Girault, J.-P.; Lafont, R. Ecdysteroids from Chenopodium Quinoa Willd., an Ancient Andean Crop of High Nutritional Value. *Food Chem.* **2011**, *125*, 1226–1234, doi:10.1016/j.foodchem.2010.10.039.
79. Nsimba, R.Y.; Kikuzaki, H.; Konishi, Y. Ecdysteroids Act as Inhibitors of Calf Skin Collagenase and Oxidative Stress. *J. Biochem. Mol. Toxicol.* **2008**, *22*, 240–250, doi:10.1002/jbt.20234.
80. Villacrés, E.; Pástor, G.; Quelal, M.B.; Zambrano, I.; Morales, S.H. Effect of Processing on the Content of Fatty Acids, Tocopherols and Sterols in the Oils of Quinoa (Chenopodium Quinoa Willd), Lupine (Lupinus Mutabilis Sweet), Amaranth (Amaranthus Caudatus L.) and Sangorache (Amaranthus Quitensis L.). **2013**.
81. Dini, A.; Rastrelli, L.; Saturnino, P.; Schettino, O. A Compositional Study of Chenopodium Quinoa Seeds. *Food Nahr.* **1992**, *36*, 400–404, doi:10.1002/food.19920360412.

82. Chai, X.-Y.; Xu, Z.-R.; Bai, C.-C.; Zhou, F.-R.; Tu, P.-F. A New Seco-Friedelolactone Acid from the Bark and Twigs of Itoa Orientalis. *Fitoterapia* **2009**, *80*, 408–410, doi:10.1016/j.fitote.2009.05.014.
83. Batta, A.K.; Xu, G.; Honda, A.; Miyazaki, T.; Salen, G. Stigmasterol Reduces Plasma Cholesterol Levels and Inhibits Hepatic Synthesis and Intestinal Absorption in the Rat. *Metabolism.* **2006**, *55*, 292–299, doi:10.1016/j.metabol.2005.08.024.
84. Huang, J.G.; Zhou, L.J.; Xu, H.H.; Li, W.O. Insecticidal and Cytotoxic Activities of Extracts of Cacalia Tangutica and Its Two Active Ingredients against Musca Domestica and Aedes Albopictus. *J. Econ. Entomol.* **2009**, *102*, 1444–1447, doi:10.1603/029.102.0407.
85. Escribano, J.; Cabanes, J.; Jiménez-Atiénzar, M.; Ibañez-Tremolada, M.; Gómez-Pando, L.R.; García-Carmona, F.; Gandía-Herrero, F. Characterization of Betalains, Saponins and Antioxidant Power in Differently Colored Quinoa (Chenopodium Quinoa) Varieties. *Food Chem.* **2017**, *234*, 285–294, doi:10.1016/j.foodchem.2017.04.187.
86. Esatbeyoglu, T.; Wagner, A.E.; Motafakkerazad, R.; Nakajima, Y.; Matsugo, S.; Rimbach, G. Free Radical Scavenging and Antioxidant Activity of Betanin: Electron Spin Resonance Spectroscopy Studies and Studies in Cultured Cells. *Food Chem. Toxicol.* **2014**, *73*, 119–126, doi:10.1016/j.fct.2014.08.007.
87. Hirakawa, N.; Okauchi, R.; Miura, Y.; Yagasaki, K. Anti-Invasive Activity of Niacin and Trigonelline against Cancer Cells. *Biosci. Biotechnol. Biochem.* **2005**, *69*, 653–658, doi:10.1271/bbb.69.653.
88. Shah, S.; Bodhankar, S.; Bhonde, R.; Mohan, V. Hypoglycemic Activity of Combination of Active Ingredients from Trigonella Foenumgraecum in Alloxan Induced Diabetic Mice. *Pharmacologyonline* **2006**, *1*, 65–82.
89. Oboh, G.; Akinyemi, A.J.; Adeleye, B.; Oyeleye, S.I.; Ogunsuyi, O.B.; Ademosun, A.O.; Ademiluyi, A.O.; Boligon, A.A. Polyphenolic Compositions and in Vitro Angiotensin-I-Converting Enzyme Inhibitory Properties of Common Green Leafy Vegetables: A Comparative Study. *Food Sci. Biotechnol.* **2016**, *25*, 1243–1249, doi:10.1007/s10068-016-0197-1.
90. Diao, M.; Liang, Y.; Zhao, J.; Zhang, J.; Zhang, T. Complexation of Ellagic Acid with α-Lactalbumin and Its Antioxidant Property. *Food Chem.* **2021**, *372*, 131307, doi:10.1016/j.foodchem.2021.131307.
91. Peng, Y.; Bishop, K.S.; Ferguson, L.R.; Quek, S.Y. Phenolic-Rich Feijoa Extracts from Flesh, Peel and Whole Fruit Activate Apoptosis Pathways in the LNCaP Cell Line. *Food Chem.* **2022**, *383*, 132285, doi:10.1016/j.foodchem.2022.132285.
92. Bidanchi, R.M.; Lalrindika, L.; Khushboo, M.; Bhanushree, B.; Dinata, R.; Das, M.; Nisa, N.; Lalrinzuali, S.; Manikandan, B.; Saeed-Ahmed, L.; et al. Antioxidative, Anti-Inflammatory and Anti-Apoptotic Action of Ellagic Acid against Lead Acetate Induced Testicular and Hepato-Renal Oxidative Damages and Pathophysiological Changes in Male Long Evans Rats. *Environ. Pollut.* **2022**, *302*, 119048, doi:10.1016/j.envpol.2022.119048.
93. Schröter, D.; Neugart, S.; Schreiner, M.; Grune, T.; Rohn, S.; Ott, C. Amaranth's 2-Caffeoylisocitric Acid—An Anti-Inflammatory Caffeic Acid Derivative That Impairs NF-κB Signaling in LPS-Challenged RAW 264.7 Macrophages. *Nutrients* **2019**, *11*, 571, doi:10.3390/nu11030571.
94. Medoua, G.N.; Oldewage-Theron, W.H. Effect of Drying and Cooking on Nutritional Value and Antioxidant Capacity of Morogo (Amaranthus Hybridus) a Traditional Leafy Vegetable Grown in South Africa. *J. Food Sci. Technol.* **2014**, *51*, 736–742, doi:10.1007/s13197-011-0560-4.
95. Neri, T.S.; Silva, K.W.L.; Maior, L.P.S.; Oliveira-Silva, S.K.; Azevedo, P.V.M.; Gomes, D.C.S.; Souza, M.A.; Pavão, J.M.S.J.; Costa, J.G.; Cunha, A.L.; et al. Phytochemical Characterization, Antioxidant Potential and Antibacterial Activity of the Croton Argyrophylloides Muell. Arg. (Euphorbiaceae). *Braz. J. Biol.* **2023**, *83*, e236649, doi:10.1590/1519-6984.236649.
96. Kalinova, J.; Dadakova, E. Rutin and Total Quercetin Content in Amaranth (Amaranthus Spp.). *Plant Foods Hum. Nutr.* **2008**, *64*, 68, doi:10.1007/s11130-008-0104-x.

97. Nayaka, H.B.; Londonkar, R.L.; Umesh, M.K.; Tukappa, A. Antibacterial Attributes of Apigenin, Isolated from *Portulaca Oleracea L. Int. J. Bacteriol.* **2014**, *2014*, e175851, doi:10.1155/2014/175851.
98. Ejoh, S.I.; Wireko-Manu, F.D.; Page, D.; Renard, C.M. Traditional Green Leafy Vegetables as Underutilised Sources of Micronutrients in a Rural Farming Community in South-West Nigeria I: Estimation of Vitamin C, Carotenoids and Mineral Contents. *South Afr. J. Clin. Nutr.* **2021**, *34*, 40–45, doi:10.1080/16070658.2019.1652963.
99. Duarte-Almeida, J.M.; Santos, R.J. dos; Genovese, M.I.; Lajolo, F.M. Avaliação da atividade antioxidante utilizando sistema beta-caroteno/ácido linoléico e método de seqüestro de radicais DPPH•. *Food Sci. Technol.* **2006**, *26*, 446–452, doi:10.1590/S0101-20612006000200031.
100. Li, Y.; Hu, K.; Huang, C.; Hu, Y.; Ji, H.; Liu, S.; Gao, J. Improvement of Solubility, Stability and Antioxidant Activity of Carotenoids Using Deep Eutectic Solvent-Based Microemulsions. *Colloids Surf. B Biointerfaces* **2022**, *217*, 112591, doi:10.1016/j.colsurfb.2022.112591.
101. Nana, F.W.; Hilou, A.; Millogo, J.F.; Nacoulma, O.G. Phytochemical Composition, Antioxidant and Xanthine Oxidase Inhibitory Activities of Amaranthus Cruentus L. and Amaranthus Hybridus L. Extracts. *Pharmaceuticals* **2012**, *5*, 613–628, doi:10.3390/ph5060613.
102. Tang, Y.; Tsao, R. Phytochemicals in Quinoa and Amaranth Grains and Their Antioxidant, Anti-inflammatory, and Potential Health Beneficial Effects: A Review. *Mol. Nutr. Food Res.* **2017**, *61*, 1600767, doi:10.1002/mnfr.201600767.
